# Driving chemical reactions with polariton condensates

**DOI:** 10.1038/s41467-022-29290-9

**Published:** 2022-03-28

**Authors:** Sindhana Pannir-Sivajothi, Jorge A. Campos-Gonzalez-Angulo, Luis A. Martínez-Martínez, Shubham Sinha, Joel Yuen-Zhou

**Affiliations:** 1 Department of Chemistry and Biochemistry, University of California San Diego, La Jolla, CA 92093 USA; 2 Department of Mathematics, University of California San Diego, La Jolla, CA 92093 USA

**Keywords:** Chemical physics, Theoretical chemistry, Reaction kinetics and dynamics, Chemical physics

## Abstract

When molecular transitions strongly couple to photon modes, they form hybrid light-matter modes called polaritons. Collective vibrational strong coupling is a promising avenue for control of chemistry, but this can be deterred by the large number of quasi-degenerate dark modes. The macroscopic occupation of a single polariton mode by excitations, as observed in Bose-Einstein condensation, offers promise for overcoming this issue. Here we theoretically investigate the effect of vibrational polariton condensation on the kinetics of electron transfer processes. Compared with excitation with infrared laser sources, the vibrational polariton condensate changes the reaction yield significantly at room temperature due to additional channels with reduced activation barriers resulting from the large accumulation of energy in the lower polariton, and the many modes available for energy redistribution during the reaction. Our results offer tantalizing opportunities to use condensates for driving chemical reactions, kinetically bypassing usual constraints of fast intramolecular vibrational redistribution in condensed phase.

## Abstract (web summary)

Molecules that collectively exhibit laser-like phenomena at room temperature offer exciting prospects for the future of chemical synthesis. Here, the authors study hybrid light-molecule systems that show such a laser-like state known as a vibrational polariton condensate, and theoretically observe a large effect of this state on chemical reactivity at room temperature.

## Introduction

Light and matter couple strongly when a large number of molecules are placed within optical cavities that confine light^1–3^. As a result, hybrid light-matter excitations called polaritons form when a collective molecular transition and a photon mode coherently exchange energy faster than the individual decay from each component. Light-matter strong coupling (SC) opens up a new path to modify material properties by controlling their electromagnetic environment^4,5^. For instance, vibrational strong coupling (VSC), where an infrared cavity mode couples to an ensemble of localized molecular vibrations in a film or solution, influences chemical reactivity even without external pumping^6,7^. However, the microscopic mechanism for modification of molecular processes through hybridization with light is still poorly understood^8–10^, since it could be limited by the presence of a large number of quasi-degenerate dark modes that do not possess any photonic character and are likely to behave similarly to uncoupled molecules^10^.

A Bose–Einstein condensate of polaritons^11^ offers a solution to this problem since the macroscopic occupation of polaritonic states enhances the effects from SC. In the last decade, Bose–Einstein condensation has been demonstrated in several organic exciton-polariton systems at room temperature^12–15^. Recently, organic polariton condensates were used to build polariton transistors^16^, and theoretical predictions suggest they may also modify incoherent charge transport^17^. Interestingly, the consequences of polariton condensation on chemical reactivity have not been addressed in the literature prior to the present study.

Ideas of using Bose–Einstein condensates of molecules in chemistry have been previously proposed, but they require ultracold temperatures due to the large mass of the condensing entities^18,19^. The low effective mass that polaritons inherit from their photonic component, along with the large binding energy of Frenkel excitons, enables room-temperature condensation^20^. The partly photonic character of polaritons also offers additional benefits such as delocalization and remote action for manipulating chemistry^21^.

**Fig. 1:**
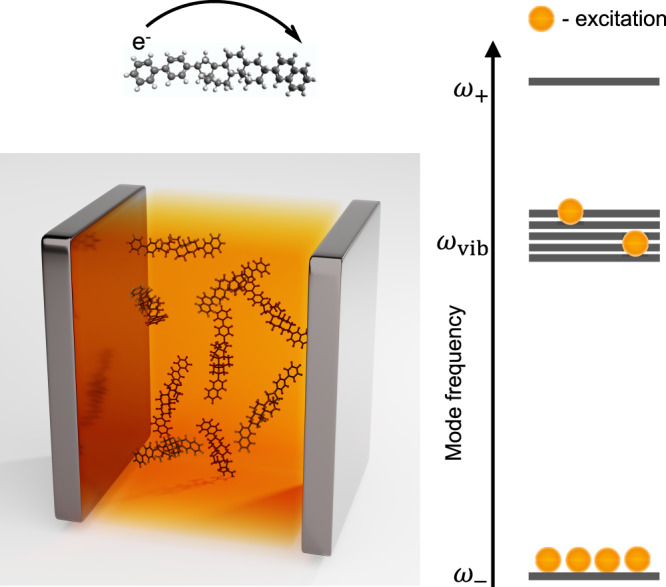
Vibrational polariton condensate. A large number of molecules are placed inside an optical cavity where their vibrations strongly couple to the cavity mode. The system is constantly pumped to create a polariton condensate and the right side of the figure depicts the occupation of different modes under condensation (frequencies of the upper polariton, dark modes, and lower polariton are *ω*_+_, *ω*_vib_, and *ω*_−_, respectively). The rate of intramolecular electron transfer under vibrational polariton condensation is investigated.

Here, we investigate the effect of polariton condensation on electron transfer (ET) (Fig. 1). While the reaction yield under infrared laser excitation, without SC, already differs from that under thermal equilibrium conditions^22,23^, polariton condensation amplifies this difference by changing the activation barrier for the forward and backward reactions unevenly, tilting the equilibrium towards either reactants or products.

## Results

### Bose–Einstein condensation of vibrational polaritons

Bose–Einstein condensation of vibrational polaritons has not been experimentally achieved yet; however, as we shall argue, there are compelling reasons to believe that they are presently within reach. Most theoretical investigations on polariton condensation with organic microcavities involve systems under electronic strong coupling (ESC)^24,25^; polariton condensation under VSC requires a separate treatment due to the difference in energy scales and the involved relaxation pathways^26^. While typical bare exciton energies range from 2–3 eV with Rabi splitting ~200 meV under ESC, the bare frequency of vibrations is 100–300 meV with Rabi splitting ~20−40 meV under VSC. Since the Rabi splitting is of the order of *k*_B_*T* at room temperature, thermal effects are crucial for vibrational polaritons. Under ESC, polariton relaxation is assisted by high-frequency intramolecular vibrations^27^, whereas, under VSC, low-frequency solvent modes play a key role in this process^28,29^, similar to what happens in THz phonon Fröhlich condensation in biomolecules^30,31^.

We model the polariton system as a set of *N* vibrational modes (${\hat{a}}_{{{{{{{{\rm{vib}}}}}}}},j}$), with frequency *ω*_vib_, strongly coupled to a single photon mode (${\hat{a}}_{{{{{{{{\rm{ph}}}}}}}}}$) with frequency *ω*_ph_. In the Hamiltonian of the system,

1 $$\hat{H}=	 \;\hslash {\omega }_{{{{{{{{\rm{ph}}}}}}}}}\left({\hat{a}}_{{{{{{{{\rm{ph}}}}}}}}}^{{{{\dagger}}} }{\hat{a}}_{{{{{{{{\rm{ph}}}}}}}}}+\frac{1}{2}\right)+\hslash {\omega }_{{{{{{{{\rm{vib}}}}}}}}}\mathop{\sum }\limits_{j=1}^{N}\left({\hat{a}}_{{{{{{{{\rm{vib}}}}}}}},j}^{{{{\dagger}}} }{\hat{a}}_{{{{{{{{\rm{vib}}}}}}}},j}+\frac{1}{2}\right)\\ 	+\mathop{\sum }\limits_{j = 1}^{N}\hslash g\left({\hat{a}}_{{{{{{{{\rm{vib}}}}}}}},j}^{{{{\dagger}}} }{\hat{a}}_{{{{{{{{\rm{ph}}}}}}}}}+{\hat{a}}_{{{{{{{{\rm{ph}}}}}}}}}^{{{{\dagger}}} }{\hat{a}}_{{{{{{{{\rm{vib}}}}}}}},j}\right),$$

we have applied the rotating wave approximation. Upon diagonalizing this Hamiltonian, we get normal modes: lower and upper polaritons, and *N* − 1 dark mode with frequencies *ω*_−_, *ω*_+_, and ${\omega }_{{{{{{{{\rm{D}}}}}}}}}^{k}$, respectively:

2 $${\omega }_{\pm } 	={\omega }_{{{{{{{{\rm{vib}}}}}}}}}+\frac{{{\Delta }}\pm {{\Omega }}}{2},\\ {\omega }_{{{{{{{{\rm{D}}}}}}}}}^{k} 	={\omega }_{{{{{{{{\rm{vib}}}}}}}}}\qquad \qquad 2\le k\le N,$$

where ${{\Omega }}=\sqrt{{{{\Delta }}}^{2}+4{g}^{2}N}$ is the Rabi splitting and Δ = *ω*_ph_ − *ω*_vib_ the detuning between cavity and molecular vibrations. To model polariton population dynamics, we use Boltzmann rate equations where the polariton system is weakly coupled to a low-frequency solvent bath, which enables scattering between modes^32^. These rate equations also account for final-state stimulation,

3 $$\frac{d{n}_{i}}{dt}=\mathop{\sum}\limits_{j}\left({W}_{ij}{n}_{j}(1+{n}_{i})-{W}_{ji}(1+{n}_{j}){n}_{i}\right)-{\gamma }_{i}{n}_{i}+{P}_{i},$$

where *n*_*i*_ is the population, *γ*_*i*_ is the decay rate and *P*_*i*_ is the external pumping rate of the *i*^*th*^ mode. The scattering rate from mode *j* to *i*, *W*_*ij*_, satisfies detailed balance: ${W}_{ij}/{W}_{ji}={e}^{-\beta ({\epsilon }_{i}-{\epsilon }_{j})}$. Here, *β* = 1/(*k*_B_*T*), *k*_*B*_ is the Boltzmann constant, *T* is the temperature, and *ϵ*_*i*_ = *ℏω*_*i*_ where *ω*_*i*_ is the frequency of the *i*^*th*^ mode. The decay from different modes is ${\gamma }_{i}=| {c}_{{{{{{{{\rm{vib}}}}}}}}}^{i}{| }^{2}{{{\Gamma }}}_{\downarrow }+| {c}_{{{{{{{{\rm{ph}}}}}}}}}^{i}{| }^{2}\kappa$, where $| {c}_{{{{{{{{\rm{vib}}}}}}}}}^{i}{| }^{2}$ and $| {c}_{{{{{{{{\rm{ph}}}}}}}}}^{i}{| }^{2}$ are the molecular and photon fraction, respectively, Γ_*↓*_ is the decay rate of the molecular vibrations, and *κ* is the cavity leakage rate.

Two factors play a determining role in the condensation threshold: (i) the rate of scattering between polariton and dark modes relative to losses from the system, i.e., the rate of thermalization and (ii) the abundance of modes close in energy to the condensing mode^33^. For all calculations, we assume fast thermalization. As mentioned in (ii), the presence of many modes close to the lower polariton would deter condensation by distributing the energy pumped into the system among all these modes. Thus, the energetic proximity between the dark state manifold, which has a large density of states (DOS), and the lower polariton pose one of the biggest challenges for polariton condensation under VSC.

**Fig. 2:**
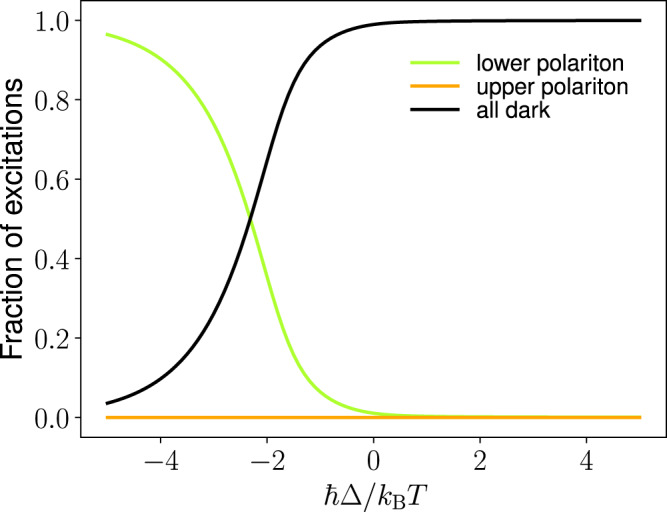
Polariton condensation transition. Fraction of excitations in different modes as a function of cavity detuning ℏΔ = ℏω_ph_ − ℏ*ω*_vib_ while keeping the pumping rate *P*_−_ fixed. The black curve includes the excitations in all dark modes taken together while the green and orange curves show the fraction excitations in the lower and upper polariton, respectively. The condensation transition takes place close to ℏΔ ≈ −1.5*k*_B_*T*. Here, the lower polariton is pumped with rate *P*_−_ = 0.16*N*Γ_*↓*_, the light-matter coupling $2\hslash g\sqrt{N}=0.72{k}_{{{{{{{{\rm{B}}}}}}}}}T$, the temperature *k*_B_*T* = 0.1389ℏ*ω*_vib_ (*T* = 298 K when ℏ*ω*_vib_ = 185 meV), number of molecules *N* = 10^7^ and cavity leakage rate *κ* = Γ_*↓*_.

The distribution of excitations between the polariton and dark modes is shown in Fig. 2 for different detunings and we observe a condensation transition at *ℏ*Δ ≈ −1.5*k*_B_*T* (see Supplementary Note 2 for details). Above the condensation threshold, a large fraction of excitations resides in the lower polariton $\frac{{n}_{-}}{(\mathop{\sum }\nolimits_{k = 2}^{\infty }{n}_{{{{{{{{\rm{D}}}}}}}}}^{k})}\gg \frac{1}{(N-1){e}^{-\beta \hslash ({{\Omega }}-{{\Delta }})/2}}$.

**Fig. 3:**
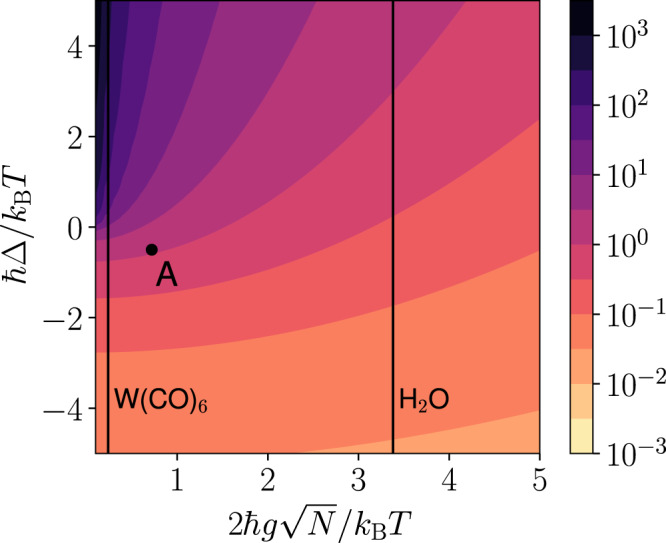
Polariton condensation threshold. Numerically obtained average population per molecule at the condensation threshold $\bar{n}={P}_{{{{{{{{\rm{th}}}}}}}}}/N{{{\Gamma }}}_{\downarrow }$ (10% of the excitations are in the lower polariton), for a range of light-matter coupling strengths $2\hslash g\sqrt{N}$ and cavity detunings ℏΔ = ℏ*ω*_ph_ − ℏ*ω*_vib_. In the black and purple regions of the plot (Δ > 0 and $2\hslash g\sqrt{N}/{k}_{{{{{{{{\rm{B}}}}}}}}}T \, < \, 2$), the threshold for condensation is high, $\bar{n}\gg 1$, and polariton condensation is difficult to achieve experimentally. The threshold for condensation is much lower, $\bar{n} \, < \, 0.1$, for the lighter colored (yellow, orange) regions. In our plot above only the upper polariton is pumped and we use cavity leakage rate *κ* = Γ_*↓*_. The vertical lines correspond to $2\hslash g\sqrt{N}/{k}_{{{{{{{{\rm{B}}}}}}}}}T$ at room temperature for H_2_O ($2\hslash g\sqrt{N}\approx 700{{{{{{{{\rm{cm}}}}}}}}}^{-1}$) and W(CO)_6_ ($2\hslash g\sqrt{N}\approx 50{{{{{{{{\rm{cm}}}}}}}}}^{-1}$). Calculations in Figs. 4–6 are presented for the conditions in point A.

The average population per molecule at the condensation threshold $\bar{n}={P}_{{{{{{{{\rm{th}}}}}}}}}/N{{{\Gamma }}}_{\downarrow }$ is a good measure of the feasibility of vibrational polariton condensation. For instance, demanding population inversion, $\bar{n} \, > \, 0.5$, would be experimentally difficult to achieve in general. In Fig. 3, we plot $\bar{n}$ for different light-matter coupling strengths, $2\hslash g\sqrt{N}$, and detunings, *ℏ*Δ. Here, we numerically obtain *P*_th_ as the pumping rate when 10% of the excitations are in the lower polariton. The threshold obtained this way closely corresponds with the theoretical condition for condensation

4 $${\bar{n}}_{{{{{{{{\rm{D}}}}}}}}}^{k} \, > \,{n}_{{{{{{{{\rm{solvent}}}}}}}}}\left(\frac{\hslash ({{\Omega }}-{{\Delta }})}{2}\right),$$

where, ${\bar{n}}_{{{{{{{{\rm{D}}}}}}}}}^{k}=\frac{1}{N-1}\mathop{\sum }\nolimits_{k = 2}^{N}{n}_{{{{{{{{\rm{D}}}}}}}}}^{k}$ is the average occupation of a dark mode, and *n*_solvent_(*E*) is the Bose–Einstein population of a solvent mode with energy *E* at room temperature *T*_room_^33^. The energy difference between the lower polariton and the dark state reservoir *ℏ*(Ω − Δ)/2 determines the condensation threshold. From Fig. 3 we see that vibrational polariton condensation is feasible for water even at room temperature for up to zero detuning.

Our model does not include disorder; as a result, all dark modes are degenerate at frequency *ω*_vib_, but in experimental systems, inhomogeneous broadening of transitions can lead to a nonzero density of dark states even at the bottom of the lower polariton branch^34^. This fact will affect the condensation threshold and should be considered in the future while looking for experimental systems that can demonstrate vibrational polariton condensation. Stimulating the lower polariton directly by shining a resonant laser on it^16^ or using a Raman scattering scheme^35^ can help overcome this issue by dynamically lowering the condensation threshold.

### Chemical reactions and vibrational polariton condensation

Electron transfer has been theoretically studied under both ESC^36,37^ and VSC^38,39^. Here, we look at how vibrational polariton condensation affects the rate of intramolecular nonadiabatic electron transfer using the VSC version^38^ of the Marcus–Levich–Jortner (MLJ) model^40–42^.

Our system consists of *N* molecules placed inside an optical cavity supporting a single photon mode with a bosonic operator ${\hat{a}}_{{{{{{{{\rm{ph}}}}}}}}}$ and frequency *ω*_ph_. The molecules can be in the reactant R or product P electronic state; for the *i*^*th*^ molecule, these states are denoted by $\left|{{{{{{{{\rm{R}}}}}}}}}_{i}\right\rangle$ and $\left|{{{{{{{{\rm{P}}}}}}}}}_{i}\right\rangle$, respectively. Each electronic state is dressed with a high-frequency intramolecular vibrational mode with a bosonic operator ${\hat{a}}_{x,i}$ and frequency *ω*_vib_ where *x* = R, P; this mode couples to the photon mode. The equilibrium geometry of this vibrational mode depends on the electronic state according to,

5 $${\hat{a}}_{{{{{{{{\rm{R}}}}}}}},i}={\hat{D}}_{i}^{{{{\dagger}}} }{\hat{a}}_{{{{{{{{\rm{P}}}}}}}},i}{\hat{D}}_{i},$$

where ${\hat{D}}_{i}=\exp (({\hat{a}}_{{{{{{{{\rm{P}}}}}}}},i}^{{{{\dagger}}} }-{\hat{a}}_{{{{{{{{\rm{P}}}}}}}},i})\sqrt{S})$ and *S* is the Huang–Rhys factor.

Apart from the intramolecular vibrations, an effective low-frequency solvent mode surrounding each molecule facilitates ET. It is treated classically, with **q**_S,*i*_ and **p**_S,*i*_ being its position and momentum.

The Hamiltonian $\hat{H}$ for the full system is a generalization of Eq. (1) to account for the chemical reaction,

6 $$\hat{H}={\hat{H}}_{0}+{\hat{V}}_{{{{{{{{\rm{react}}}}}}}}},$$

and

7 $${\hat{H}}_{0}	={\hat{H}}_{{{{{{{{\rm{ph}}}}}}}}}+\mathop{\sum }\limits_{i=1}^{N}\mathop{\sum}\limits_{x={{{{{{{\rm{R}}}}}}}},{{{{{{{\rm{P}}}}}}}}}({\hat{H}}_{x,i}+{\hat{V}}_{x,i})\left|{x}_{i}\right\rangle \left\langle {x}_{i}\right|,\\ {\hat{V}}_{{{{{{{{\rm{react}}}}}}}}}	=\mathop{\sum }\limits_{i=1}^{N}{J}_{{{{{{{{\rm{R}}}}}}}}{{{{{{{\rm{P}}}}}}}}}\left(\left|{{{{{{{{\rm{R}}}}}}}}}_{i}\right\rangle \left\langle {{{{{{{{\rm{P}}}}}}}}}_{i}\right|+\left|{{{{{{{{\rm{P}}}}}}}}}_{i}\right\rangle \left\langle {{{{{{{{\rm{R}}}}}}}}}_{i}\right|\right).$$

where ${\hat{H}}_{0}$ describes the photon (${\hat{H}}_{{{{{{{{\rm{ph}}}}}}}}}$), intramolecular vibrations and solvent modes of the *i*th molecule (${\hat{H}}_{x,i}$), and light-matter couplings (${\hat{V}}_{x,i}$). The diabatic coupling ${\hat{V}}_{{{{{{{{\rm{react}}}}}}}}}$ is a perturbation that couples R and P electronic states with coupling strength *J*_RP_. We have taken the dipole moment to be zero when the vibrational coordinate is in its equilibrium position in both R and P electronic states. Relaxing this assumption will add terms of the form ${c}_{x}({\hat{a}}_{{{{{{{{\rm{ph}}}}}}}}}+{\hat{a}}_{{{{{{{{\rm{ph}}}}}}}}})\left|{x}_{i}\right\rangle \left\langle {x}_{i}\right|$ to the Hamiltonian and will not affect the reaction rates calculated.

8 $${\hat{H}}_{{{{{{{{\rm{ph}}}}}}}}} 	=\hslash {\omega }_{{{{{{{{\rm{ph}}}}}}}}}\left({\hat{a}}_{{{{{{{{\rm{ph}}}}}}}}}^{{{{\dagger}}} }{\hat{a}}_{{{{{{{{\rm{ph}}}}}}}}}+\frac{1}{2}\right),\\ {\hat{H}}_{{{{{{{{\rm{R}}}}}}}},i} 	=\hslash {\omega }_{{{{{{{{\rm{vib}}}}}}}}}\left({\hat{a}}_{{{{{{{{\rm{R}}}}}}}},i}^{{{{\dagger}}} }{\hat{a}}_{{{{{{{{\rm{R}}}}}}}},i}+\frac{1}{2}\right)+\frac{1}{2}\hslash {\omega }_{{{{{{{{\rm{S}}}}}}}}}\left(| {{{{{{{{\bf{p}}}}}}}}}_{{{{{{{{\rm{S}}}}}}}},i}{| }^{2}+| {{{{{{{{\bf{q}}}}}}}}}_{{{{{{{{\rm{S}}}}}}}},i}+{{{{{{{{\bf{d}}}}}}}}}_{{{{{{{{\rm{S}}}}}}}}}{| }^{2}\right),\\ {\hat{H}}_{{{{{{{{\rm{P}}}}}}}},i} 	=\hslash {\omega }_{{{{{{{{\rm{vib}}}}}}}}}\left({\hat{a}}_{{{{{{{{\rm{P}}}}}}}},i}^{{{{\dagger}}} }{\hat{a}}_{{{{{{{{\rm{P}}}}}}}},i}+\frac{1}{2}\right)+\frac{1}{2}\hslash {\omega }_{{{{{{{{\rm{S}}}}}}}}}\left(| {{{{{{{{\bf{p}}}}}}}}}_{{{{{{{{\rm{S}}}}}}}},i}{| }^{2}+| {{{{{{{{\bf{q}}}}}}}}}_{{{{{{{{\rm{S}}}}}}}},i}{| }^{2}\right)+{{\Delta }}G,\\ {\hat{V}}_{x,i} 	=\hslash {g}_{x}({\hat{a}}_{x,i}^{{{{\dagger}}} }{\hat{a}}_{{{{{{{{\rm{ph}}}}}}}}}+{\hat{a}}_{{{{{{{{\rm{ph}}}}}}}}}^{{{{\dagger}}} }{\hat{a}}_{x,i}),$$

where Δ*G* is the free-energy difference of each individual molecule reaction.

We construct potential energy surfaces (PES) by parametrically diagonalizing ${\hat{H}}_{0}$ as a function of the solvent coordinate **q**_S,*i*_. The operators ${\hat{N}}_{{{{{{{{\rm{R}}}}}}}}}=\mathop{\sum }\nolimits_{i = 1}^{N}\left|{{{{{{{{\rm{R}}}}}}}}}_{i}\right\rangle \left\langle {{{{{{{{\rm{R}}}}}}}}}_{i}\right|$ and ${\hat{N}}_{{{{{{{{\rm{P}}}}}}}}}=\mathop{\sum }\nolimits_{i = 1}^{N}\left|{{{{{{{{\rm{P}}}}}}}}}_{i}\right\rangle \left\langle {{{{{{{{\rm{P}}}}}}}}}_{i}\right|$ commute with *H*_0_ and correspond to the number of R and P molecules, respectively. While dynamics under ${\hat{H}}_{0}$ conserves *N*_R_, *N*_P_, the effect of ${\hat{V}}_{{{{{{{{\rm{react}}}}}}}}}$ is to induce reactive transitions that modify those quantities while keeping *N* = *N*_R_ + *N*_P_ constant. We assign the label 1 ≤ *i* ≤ *N*_R_ to R molecules, and *N*_R_ + 1 ≤ *i* ≤ *N* to P molecules. We also reorganize the intramolecular vibrations into a single bright mode,

9 $${\hat{a}}_{{{{{{{{\rm{B}}}}}}}}({N}_{{{{{{{{\rm{R}}}}}}}}},{N}_{{{{{{{{\rm{P}}}}}}}}})}=\frac{1}{\sqrt{{g}_{{{{{{{{\rm{R}}}}}}}}}^{2}{N}_{{{{{{{{\rm{R}}}}}}}}}+{g}_{{{{{{{{\rm{P}}}}}}}}}^{2}{N}_{{{{{{{{\rm{P}}}}}}}}}}}\left({g}_{{{{{{{{\rm{R}}}}}}}}}\mathop{\sum }\limits_{i=1}^{{N}_{{{{{{{{\rm{R}}}}}}}}}}{\hat{a}}_{{{{{{{{\rm{R}}}}}}}},i}+{g}_{{{{{{{{\rm{P}}}}}}}}}\mathop{\sum }\limits_{i={N}_{{{{{{{{\rm{R}}}}}}}}}+1}^{N}{\hat{a}}_{{{{{{{{\rm{P}}}}}}}},i}\right),$$

that possesses the correct symmetry to couple with light and *N* − 1 dark modes (D_*k*_),

10 $${\hat{a}}_{{{{{{{{\rm{D}}}}}}}}({N}_{{{{{{{{\rm{R}}}}}}}}},{N}_{{{{{{{{\rm{P}}}}}}}}})}^{k}=\mathop{\sum }\limits_{i=1}^{{N}_{{{{{{{{\rm{R}}}}}}}}}}{c}_{k,i}{\hat{a}}_{{{{{{{{\rm{R}}}}}}}},i}+\mathop{\sum }\limits_{i={N}_{{{{{{{{\rm{R}}}}}}}}}+1}^{N}{c}_{k,i}{\hat{a}}_{{{{{{{{\rm{P}}}}}}}},i},$$

labeled by an additional index 2 ≤ *k* ≤ *N*, which do not couple with light. The dark modes are orthogonal to the bright mode ${g}_{{{{{{{{\rm{R}}}}}}}}}\mathop{\sum }\nolimits_{i = 1}^{{N}_{{{{{{{{\rm{R}}}}}}}}}}{c}_{k,i}+{g}_{{{{{{{{\rm{P}}}}}}}}}\mathop{\sum }\nolimits_{i = {N}_{{{{{{{{\rm{R}}}}}}}}}+1}^{N}{c}_{k,i}=0$, and to each other $\mathop{\sum }\nolimits_{i = 1}^{N}{c}_{k,i}{c}_{k^{\prime} ,i}^{* }={\delta }_{k,k^{\prime} }$. Unless mentioned otherwise, the number of R and P molecules is *N*_R_ and *N*_P_, respectively, and for brevity, we will drop (*N*_R_, *N*_P_) dependence in the subscript. The bright and photon modes combine to form the upper polariton (UP) ${\hat{a}}_{+}$, and lower polariton (LP) ${\hat{a}}_{-}$, modes:

11 $${\hat{a}}_{+}	=\cos \theta {\hat{a}}_{{{{{{{{\rm{ph}}}}}}}}}+\sin \theta {\hat{a}}_{{{{{{{{\rm{B}}}}}}}}},\\ {\hat{a}}_{-}	=\sin \theta {\hat{a}}_{{{{{{{{\rm{ph}}}}}}}}}-\cos \theta {\hat{a}}_{{{{{{{{\rm{B}}}}}}}}},$$

with mixing angle,

12 $$\theta ={\tan }^{-1}\left[\frac{{{\Omega }}-{{\Delta }}}{2\sqrt{{g}_{{{{{{{{\rm{R}}}}}}}}}^{2}{N}_{{{{{{{{\rm{R}}}}}}}}}+{g}_{{{{{{{{\rm{P}}}}}}}}}^{2}{N}_{{{{{{{{\rm{P}}}}}}}}}}}\right],$$

where ${{\Omega }}=\sqrt{{{{\Delta }}}^{2}+4({g}_{{{{{{{{\rm{R}}}}}}}}}^{2}{N}_{{{{{{{{\rm{R}}}}}}}}}+{g}_{{{{{{{{\rm{P}}}}}}}}}^{2}{N}_{{{{{{{{\rm{P}}}}}}}}})}$ is the Rabi splitting, and Δ = *ω*_ph_ − *ω*_vib_ the detuning between the cavity and molecular vibrations. The eigenstates of ${\hat{H}}_{0}$ are the dark, upper, and lower polariton modes with frequencies given in Eq. (2).

According to the MLJ theory, the rate constant for ET outside of an optical cavity depends on properties of the intramolecular and solvent modes^40–42^. Under laser driving, this rate constant is,

13 $${k}_{{{{{{{{\rm{R}}}}}}}}\to {{{{{{{\rm{P}}}}}}}}}^{{{{{{{{\rm{IR}}}}}}}}}=\mathop{\sum }\limits_{n=0}^{\infty }{P}_{\bar{n}}(n){k}_{{{{{{{{\rm{R}}}}}}}}\to {{{{{{{\rm{P}}}}}}}}}(n)$$

where

14 $${k}_{{{{{{{{\rm{R}}}}}}}}\to {{{{{{{\rm{P}}}}}}}}}(n) 	=\sqrt{\frac{\pi }{{\lambda }_{{{{{{{{\rm{S}}}}}}}}}{k}_{{{{{{{{\rm{B}}}}}}}}}T}}\frac{| {J}_{{{{{{{{\rm{R}}}}}}}}{{{{{{{\rm{P}}}}}}}}}{| }^{2}}{\hslash }\mathop{\sum }\limits_{f=-n}^{\infty }| \left\langle n| n+f\right\rangle ^{\prime} {| }^{2}\exp \left(-\frac{{E}_{f}^{{{{\ddagger}}} }}{{k}_{{{{{{{{\rm{B}}}}}}}}}T}\right),\\ {P}_{\bar{n}}(n) 	={e}^{-\bar{n}}\frac{{\bar{n}}^{n}}{n!},\\ {E}_{f}^{{{{\ddagger}}} } 	=\frac{{({{\Delta }}G+{\lambda }_{{{{{{{{\rm{S}}}}}}}}}+f\hslash {\omega }_{{{{{{{{\rm{vib}}}}}}}}})}^{2}}{4{\lambda }_{{{{{{{{\rm{S}}}}}}}}}},\\ \left\langle n| n+f\right\rangle ^{\prime} 	=\left\langle n\right|{\hat{D}}_{i}\left|n+f\right\rangle .$$

Here, ${P}_{\bar{n}}(n)$ is the Poisson distribution with average mode population $\bar{n}$, *λ*_S_ is the solvent reorganization energy, ${E}_{f}^{{{{\ddagger}}} }$ is the activation energy, and $| \left\langle n| n+f\right\rangle ^{\prime} {| }^{2}$ is the Franck–Condon (FC) factor, where $\left|n\right\rangle$ and $\left|n+f\right\rangle ^{\prime}$ are the intramolecular initial and final states, respectively. ${P}_{\bar{n}}(n)$ has been taken to correspond to the ideal laser-driven-damped harmonic oscillator, leading to a coherent state in the vibrational mode. The presence of anharmonic couplings would lead to intramolecular vibrational energy redistribution (IVR)^43^, reducing the value of ${P}_{\bar{n}}(n)$ for high-lying Fock states. However, as we shall see below, even under these ideal circumstances, the condensate can outcompete the laser-driven situation in terms of reactivity. We thus expect the benefits of the condensate to be enhanced when IVR processes are taken into account.

Apart from vibrations within the reacting molecule, under VSC, the ET process also depends on vibrations in all other molecules and the photon mode, and can be represented by,

15 $$\mathop{\sum }\limits_{k=2}^{N}{{{{{{{{\rm{D}}}}}}}}}_{k}+{{{{{{{\rm{LP}}}}}}}}+{{{{{{{\rm{UP}}}}}}}}\to \mathop{\sum }\limits_{k=2}^{N}{{{{{{{\rm{D}}}}}}}}_{k}^{\prime} +{{{{{{{\rm{LP}}}}}}}}^{\prime} +{{{{{{{\rm{UP}}}}}}}}^{\prime} .$$

Here and hereafter, the primed and unprimed quantities refer to electronic states with (*N*_R_, *N*_P_) and (*N*_R_ − 1, *N*_P_ + 1) reactant-product distributions, respectively. The symmetry of the light-matter coupling allows us to use the dark state basis introduced in^44^ and^38^ to reduce the number of modes involved in the reaction from *N* + 1 to three,

16 $${{{{{{{{\rm{D}}}}}}}}}_{{{{{{{{\rm{R}}}}}}}},c}+{{{{{{{\rm{LP}}}}}}}}+{{{{{{{\rm{UP}}}}}}}}\to {{{{{{{\rm{D}}}}}}}}_{{{{{{\rm{P}}}}}},{c}}^{\prime} +{{{{{{{\rm{LP}}}}}}}}^{\prime} +{{{{{{{\rm{UP}}}}}}}}^{\prime} .$$

Here, the *c*^*th*^ molecule is reacting, while D_*x*,*c*_ and ${{{{{{{{\rm{D}}}}}}}}_{x,c}^{\prime}}$ are dark modes highly localized in it, with corresponding operators ${\hat{a}}_{{{{{{{{\rm{D}}}}}}}}}^{(R,c)}$ and ${\hat{a}}_{{{{{{{{\rm{D}}}}}}}}}^{(P,c)^{\prime} }$ (see Supplementary Note 1).

We perform all our calculations in this subsection using parameters from point A in Fig. 3 but while pumping the lower polariton. Here, *ℏ*Δ = −0.5*k*_B_*T*, $2\hslash g\sqrt{N}=0.72{k}_{{{{{{{{\rm{B}}}}}}}}}T$, *k*_B_*T* = 0.1389*ℏω*_vib_ (*T* = 298 K when *ℏω*_vib_ = 185 meV) and *N* = 10^7^; we choose pumping rate *P*_−_ = 0.08*N*Γ_*↓*_, which leads to average mode populations *N*_+_ = 0.064, *N*_−_ = 1.94 × 10^4^ and *N*_D_ = 0.078 under symmetric coupling *g*_R_ = *g*_P_ = *g*. Here, 2.4% of all excitations reside in the lower polariton. To compare the reaction rates under polariton condensation and outside the cavity under pumping, we take $\bar{n}=0.08$ in Eq. (13). Under condensation, the initial vibrational state of the system can be described by $\rho ={\sum }_{{n}_{+},{n}_{-},{n}_{{{{{{{{\rm{D}}}}}}}}}}P({n}_{+},{n}_{-},{n}_{{{{{{{{\rm{D}}}}}}}}})\left|{n}_{+},{n}_{-},{n}_{{{{{{{{\rm{D}}}}}}}}}\right\rangle \left\langle {n}_{+},{n}_{-},{n}_{{{{{{{{\rm{D}}}}}}}}}\right|$, where the entries in $\left|{n}_{+},{n}_{-},{n}_{{{{{{{{\rm{D}}}}}}}}}\right\rangle$ label number of quanta in the UP, LP, and D_R,*c*_ modes, respectively. The results from the rate equations Eq. (3) provide us only with the average steady-state mode populations, *N*_+_, *N*_−_, and *N*_D_, and not the distribution *P*(*n*_+_, *n*_−_, *n*_D_). For simplicity, we assume the semiclassical approximation $P({n}_{+},{n}_{-},{n}_{{{{{{{{\rm{D}}}}}}}}})\approx {\delta }_{{n}_{+},0}{P}_{{N}_{{{{{{{{\rm{D}}}}}}}}}}^{{{{{{{{\rm{th}}}}}}}}}({n}_{{{{{{{{\rm{D}}}}}}}}}){\delta }_{{n}_{-},{N}_{-}}$, where ${P}_{{N}_{{{{{{{{\rm{D}}}}}}}}}}^{{{{{{{{\rm{th}}}}}}}}}(n)$ is the thermal distribution with average population *N*_D_. This approximation is reasonable for populations *N*_+_ < *N*_D_ ≪ 1 ≪ *N*_−_,

17 $$\rho =\mathop{\sum}\limits_{{n}_{{{{{{{{\rm{D}}}}}}}}}}{P}_{{N}_{{{{{{{{\rm{D}}}}}}}}}}^{{{{{{{{\rm{th}}}}}}}}}({n}_{{{{{{{{\rm{D}}}}}}}}})\left|0,{N}_{-},{n}_{{{{{{{{\rm{D}}}}}}}}}\right\rangle \left\langle 0,{N}_{-},{n}_{{{{{{{{\rm{D}}}}}}}}}\right|.$$

The product vibrational states are $\left|{\nu }_{+},{\nu }_{-},{\nu }_{{{{{{{{\rm{D}}}}}}}}}\right\rangle ^{\prime}$.

We assume that cavity leakage and the rate of scattering between modes is much faster than the rate of the chemical reaction. For a cavity with ~100 ps lifetime and ET reactions with 1/*k*_R→P_ ~ 10^6^ − 10^2^ ps^45^, this assumption is valid. Therefore, if the populations of polariton modes change during the course of the reaction, they quickly reach a steady state before the next molecule reacts. Similarly, we also assume that the polariton and dark mode populations reach a steady state before the backward reaction takes place while computing the rate constant ${k}_{{{{{{{{\rm{P}}}}}}}}\to {{{{{{{\rm{R}}}}}}}}}^{{{{{{{{\rm{cond}}}}}}}}}$. Generalizing the cavity MLJ theory presented in ref. ^38^, we calculate the rate constant

18 $${k}_{{{{{{{{\rm{R}}}}}}}}\to {{{{{{{\rm{P}}}}}}}}}^{{{{{{{{\rm{cond}}}}}}}}}=\mathop{\sum }\limits_{n=0}^{\infty }{P}_{{N}_{{{{{{{{\rm{D}}}}}}}}}}^{{{{{{{{\rm{th}}}}}}}}}(n){k}_{{{{{{{{\rm{R}}}}}}}}\to {{{{{{{\rm{P}}}}}}}}}^{{{{{{{{\rm{cond}}}}}}}}}(n)$$

for the forward reaction under polariton condensation, where

19 $${k}_{{{{{{{{\rm{R}}}}}}}}\to {{{{{{{\rm{P}}}}}}}}}^{{{{{{{{\rm{cond}}}}}}}}}(n)=	 \,\sqrt{\frac{\pi }{{\lambda }_{{{{{{{{\rm{S}}}}}}}}}{k}_{{{{{{{{\rm{B}}}}}}}}}T}}\frac{| {J}_{{{{{{{{\rm{R}}}}}}}}{{{{{{{\rm{P}}}}}}}}}{| }^{2}}{\hslash }\mathop{\sum }\limits_{{\nu }_{+}=0}^{\infty }\mathop{\sum }\limits_{{\nu }_{-}=0}^{\infty }\mathop{\sum }\limits_{{\nu }_{{{{{{{{\rm{D}}}}}}}}}=0}^{\infty }{W}_{{\nu }_{+},{\nu }_{-},{\nu }_{{{{{{{{\rm{D}}}}}}}}}}^{f,n},\\ {W}_{{\nu }_{+},{\nu }_{-},{\nu }_{{{{{{{{\rm{D}}}}}}}}}}^{f,n}=	 \,| {F}_{{\nu }_{+},{\nu }_{-},{\nu }_{{{{{{{{\rm{D}}}}}}}}}}^{f,n}{| }^{2}\times \exp \left(-\frac{{E}_{{\nu }_{+},{\nu }_{-},{\nu }_{{{{{{{{\rm{D}}}}}}}}}}^{f,n{{{\ddagger}}} }}{{k}_{{{{{{{{\rm{B}}}}}}}}}T}\right).$$

The FC factor $| {F}_{{\nu }_{+},{\nu }_{-},{\nu }_{{{{{{{{\rm{D}}}}}}}}}}^{f,n}{| }^{2}=\big| \left\langle 0,{N}_{-},n| {\nu }_{+},{\nu }_{-},{\nu }_{{{{{{{{\rm{D}}}}}}}}}\right\rangle ^{\prime}\big| ^{2}$, and activation energy ${E}_{{\nu }_{+},{\nu }_{-},{\nu }_{{{{{{{{\rm{D}}}}}}}}}}^{f,n{{{\ddagger}}} }$ play an important role in determining the rate constant.

While many methods have been developed for computing multimode FC factors^46–48^, the focus has been on increasing the number of modes while keeping their occupation small. The current problem, however, offers a new technical challenge: the large occupation of LP makes the aforementioned methods computationally expensive. Instead, we draw inspiration from previous work that employs generating functions^47^ and combine those techniques with the powerful Lagrange–Bürmann formula^49^ to obtain analytical expressions for the required three-dimensional FC factors (see details in Supplementary Section S3).

The activation energies for the various channels of reactivity are,

20 $${E}_{{\nu }_{+},{\nu }_{-},{\nu }_{{{{{{{{\rm{D}}}}}}}}}}^{f,n{{{\ddagger}}} }=\frac{{({E}_{{{{{{{{\rm{P}}}}}}}}}^{{\nu }_{+},{\nu }_{-},{\nu }_{{{{{{{{\rm{D}}}}}}}}}}-{E}_{{{{{{{{\rm{R}}}}}}}}}^{0,{N}_{-},n}+{\lambda }_{{{{{{{{\rm{S}}}}}}}}})}^{2}}{4{\lambda }_{{{{{{{{\rm{S}}}}}}}}}},$$

where

21 $${E}_{{{{{{{{\rm{P}}}}}}}}}^{{\nu }_{+},{\nu }_{-},{\nu }_{{{{{{{{\rm{D}}}}}}}}}}=	 \,{{\Delta }}G+\hslash \left[{\omega}_{+}^{\prime} \left({\nu }_{+}+\frac{1}{2}\right)\right.\\ 	\left.+\,\omega_{-}^{\prime} \left({\nu }_{-}+\frac{1}{2}\right)+{\omega }_{{{{{{{{\rm{vib}}}}}}}}}\left({\nu }_{{{{{{{{\rm{D}}}}}}}}}+\frac{1}{2}\right)\right],\\ {E}_{{{{{{{{\rm{R}}}}}}}}}^{0,{N}_{-},n}=	 \,\hslash \left[{\omega }_{+}\frac{1}{2}+{\omega }_{-}\left({N}_{-}+\frac{1}{2}\right)+{\omega }_{{{{{{{{\rm{vib}}}}}}}}}\left(n+\frac{1}{2}\right)\right].$$

When condensation takes place, the number of quanta in the lower polariton *N*_−_ ~10^5^ is so large that the summation in ${k}_{{{{{{{{\rm{R}}}}}}}}\to {{{{{{{\rm{P}}}}}}}}}^{{{{{{{{\rm{cond}}}}}}}}}(n)$ becomes difficult to estimate. To simplify the computation and gain intuition, we group channels into sets with the same change in the total number of intramolecular vibrational quanta *f* = *ν*_+_ + *ν*_−_ + *ν*_D_ − *N*_−_ − *n* upon ET. The closeness in energy between PES with the same *f*, and hence similar activation barriers, is the rationale for this grouping. ${k}_{{{{{{{{\rm{R}}}}}}}}\to {{{{{{{\rm{P}}}}}}}}}^{{{{{{{{\rm{cond}}}}}}}}}(n)$ then goes from a free summation over three indices *ν*_+_, *ν*_−_ and *ν*_D_ into a summation over four indices *f*, *ν*_+_, *ν*_−_ and *ν*_D_ with the constraint *ν*_+_ + *ν*_−_ + *ν*_D_ = *N*_−_ + *n* + *f*,

22 $${k}_{{{{{{{{\rm{R}}}}}}}}\to {{{{{{{\rm{P}}}}}}}}}^{{{{{{{{\rm{cond}}}}}}}}}(n)=	 \,\sqrt{\frac{\pi }{{\lambda }_{{{{{{{{\rm{S}}}}}}}}}{k}_{{{{{{{{\rm{B}}}}}}}}}T}}\frac{| {J}_{{{{{{{{\rm{R}}}}}}}}{{{{{{{\rm{P}}}}}}}}}{| }^{2}}{\hslash }\\ 	\mathop{\sum }\limits_{f=-{N}_{-}-n}^{\infty }\mathop{\sum }\limits_{{\nu }_{+},{\nu }_{-},{\nu }_{{{{{{{{\rm{D}}}}}}}}}}^{{\nu }_{+}+{\nu }_{-}+{\nu }_{{{{{{{{\rm{D}}}}}}}}}={N}_{-}+n+f}{W}_{{\nu }_{+},{\nu }_{-},{\nu }_{{{{{{{{\rm{D}}}}}}}}}}^{f,n}.$$

**Fig. 4:**
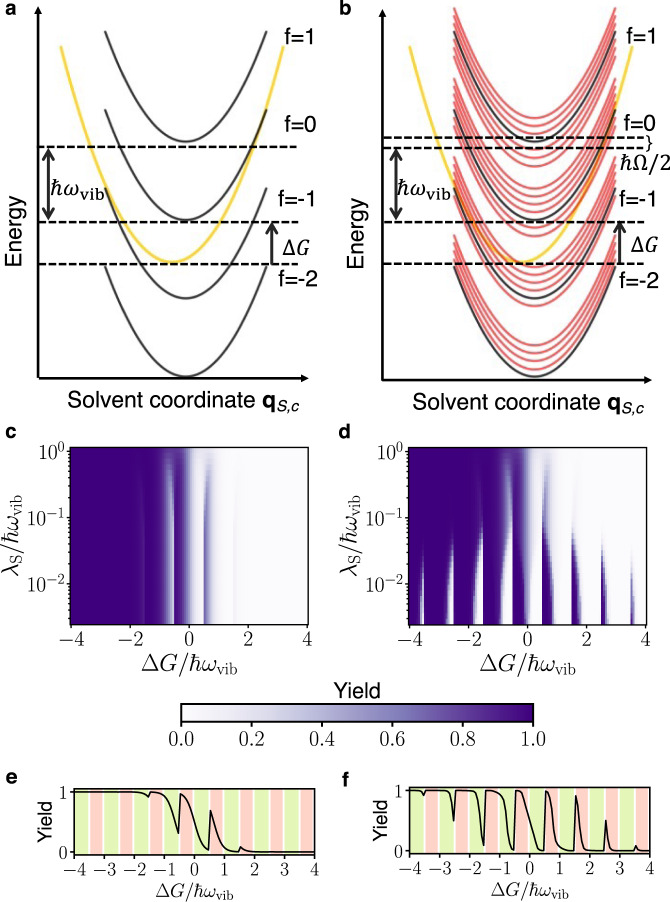
Potential energy surfaces (not to scale) and reaction yield. **a**, **c**, **e** are results for a laser-driven system without light-matter strong coupling (SC) and **b**, **d**, **f** are for the same system under SC and 2.4% of the population in the lower polariton (condensation). All these plots are for symmetric light-matter coupling *g*_R_ = *g*_P_. **a**, **b** For a clearer qualitative picture, we plot the PESs under zero detuning Δ = 0. Initial (yellow) and final (black) PESs for a molecule undergoing the forward reaction with solvent coordinate **q**_S,c_. While the energy separation between black PESs is ℏ*ω*_vib_, the condensate provides many additional final PESs (red, separated by ℏΩ/2 at resonance). **c** Reaction yield ${N}_{{{{{{{{\rm{P}}}}}}}}}^{{{{{{{{\rm{ss}}}}}}}}}/N$ at temperature *k*_B_*T* = 0.1389ℏ*ω*_vib_ (*T* = 298 K when ℏ*ω*_vib_ = 185 meV), Huang–Rhys factor *S* = 3.5, and average occupation of the intramolecular vibrational mode $\bar{n}=0.08$. **d** Reaction yield ${N}_{{{{{{{{\rm{P}}}}}}}}}^{{{{{{{{\rm{ss}}}}}}}}}/N$ with Δ = − 0.0695*ω*_vib_, $2{g}_{{{{{{{{\rm{R}}}}}}}}}\sqrt{N}=2{g}_{{{{{{{{\rm{P}}}}}}}}}\sqrt{N}=0.1{\omega }_{{{{{{{{\rm{vib}}}}}}}}}$, *P*_−_ = 0.08*N*Γ_*↓*_, *N* = 10^7^, temperature and Huang–Rhys factor are the same as **c**. The contributions of the red PESs through the condensate provide a broader tunability of reaction yields with respect to Δ*G* than under laser driving without SC. Notice that originally endergonic (exergonic) reactions in the absence of optical pumping can become exergonic (endergonic) under the featured nonequilibrium conditions. **e**, **f** A cross-section of plot (**c**, **d**) when *λ*_S_ = 10^−2^ℏ*ω*_vib_. The pink shaded regions correspond to cases where the dominant forward (backward) channel is in the inverted (normal) regime; the opposite is true for the green shaded regions. The condensate amplifies the forward (backward) reaction in the pink (green) shaded regions.

To understand the qualitative difference between reactions under polariton condensation and external pumping without SC, in Fig. 4a, b we plot the PESs (not to scale) showing the forward reaction under symmetric light-matter coupling and zero detuning. The yellow (black) parabolas in Fig. 4a, b represent PESs for a molecule in the electronic state $\left|{{{{{{{\rm{R}}}}}}}}\right\rangle$ ($\left|{{{{{{{\rm{P}}}}}}}}\right\rangle$) and vibrational state $\left|2\right\rangle$ ($\left|2+f\right\rangle ^{\prime}$) in Fig. 4a and $\left|0,{N}_{-},2\right\rangle$ ($\left|0,{N}_{-},2+f\right\rangle ^{\prime}$) in Fig. 4b. The red parabolas in Fig. 4b are additional final PESs provided by the vibrational polariton condensate (hereafter referred to solely as “condensate”) that account for all other final vibrational states $\left|{\nu }_{+},{\nu }_{-},{\nu }_{{{{{{{{\rm{D}}}}}}}}}\right\rangle ^{\prime}$.

The net rate of ET is,

23 $$\frac{d{N}_{{{{{{{{\rm{R}}}}}}}}}}{dt}=-{k}_{{{{{{{{\rm{R}}}}}}}}\to {{{{{{{\rm{P}}}}}}}}}^{z}{N}_{{{{{{{{\rm{R}}}}}}}}}+{k}_{{{{{{{{\rm{P}}}}}}}}\to {{{{{{{\rm{R}}}}}}}}}^{z}{N}_{{{{{{{{\rm{P}}}}}}}}},$$

where ${k}_{{{{{{{{\rm{R}}}}}}}}\to {{{{{{{\rm{P}}}}}}}}}^{z}$ and ${k}_{{{{{{{{\rm{P}}}}}}}}\to R}^{z}$ (*z* = IR, cond) are the rate constants for the forward and backward reactions, respectively, which are themselves functions of *N*_R_ and *N*_P_ when *g*_R_ ≠ *g*_P_. We find the steady-state solution ${N}_{{{{{{{{\rm{R}}}}}}}}}^{{{{{{{{\rm{ss}}}}}}}}}$ from this equation and compute the reaction yield ${N}_{{{{{{{{\rm{P}}}}}}}}}^{{{{{{{{\rm{ss}}}}}}}}}/N$.

The difference in yield between the condensate and bare case is particularly large when *λ*_S_ ≪ *ℏω*_vib_ < ∣Δ*G*∣ (see Fig. 4c, d for symmetric coupling *g*_R_ = *g*_P_). To understand the underlying reason, we define the dominant channel ${f}_{\min }$ as the one with minimum activation barrier outside of the cavity.

24 $$\frac{1}{{k}_{{{{{{{{\rm{B}}}}}}}}}T}\frac{d{E}_{f}^{{{{\ddagger}}} }}{df}=\frac{\hslash {\omega }_{{{{{{{{\rm{vib}}}}}}}}}}{{k}_{{{{{{{{\rm{B}}}}}}}}}T}\left(\frac{{{\Delta }}G+{\lambda }_{{{{{{{{\rm{S}}}}}}}}}+f\hslash {\omega }_{{{{{{{{\rm{vib}}}}}}}}}}{2{\lambda }_{{{{{{{{\rm{S}}}}}}}}}}\right)$$

Setting the derivative in Eq. (24) equal to zero and taking into account the discrete nature of *f*, we find the dominant channel, ${f}_{\min }=\left\lceil\frac{-{{\Delta }}G-{\lambda }_{{{{{{{{\rm{S}}}}}}}}}}{\hslash {\omega }_{{{{{{{{\rm{vib}}}}}}}}}}\right\rceil$ or $\left\lfloor\frac{-{{\Delta }}G-{\lambda }_{{{{{{{{\rm{S}}}}}}}}}}{\hslash {\omega }_{{{{{{{{\rm{vib}}}}}}}}}}\right\rfloor$. When *λ*_S_ ≪ *ℏω*_vib_, ∣Δ*G*∣, this channel contributes most to the rate constant because $\frac{1}{{k}_{{{{{{{{\rm{B}}}}}}}}}T}\left|\frac{d{E}_{f}^{{{{\ddagger}}} }}{df}\right|\gg 1$. We define Marcus normal $\frac{d{E}_{f}^{{{{\ddagger}}} }}{df}{\Big|}_{{f}_{\min }} > \, 0$ and inverted $\frac{d{E}_{f}^{{{{\ddagger}}} }}{df}{\Big|}_{{f}_{\min }} < \, 0$ regimes with respect to the dominant channel. If the dominant forward channel is in the inverted regime, the dominant backward channel (which can be found by replacing Δ*G* → − Δ*G* in Eq. (24)) will be in the normal regime when *λ*_S_ ≪ *ℏω*_vib_, ∣Δ*G*∣.

**Fig. 5:**
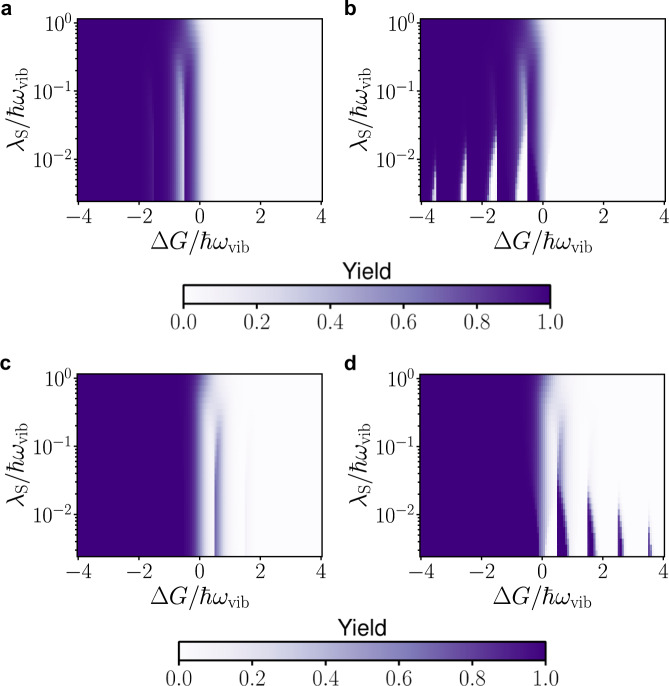
Reaction yield for asymmetric light-matter coupling. **a**, **c** The yield of the reaction when only the product (reactant) weakly couples with light. **b**, **d** Analogous plots under strong coupling $2{g}_{{{{{{{{\rm{P}}}}}}}}}\sqrt{N}=0.1{\omega }_{{{{{{{{\rm{vib}}}}}}}}}$, *g*_R_ = 0 (*g*_P_ = 0, $2{g}_{{{{{{{{\rm{R}}}}}}}}}\sqrt{N}=0.1{\omega }_{{{{{{{{\rm{vib}}}}}}}}}$). We use parameters Δ = −0.0695*ω*_vib_, *k*_B_*T* = 0.1389ℏ*ω*_vib_ (*T* = 298K when ℏ*ω*_vib_ = 185 meV), *S* = 3.5, *P*_−_ = 0.08*N*Γ_*↓*_ and *N* = 10^7^. We assume the same scattering parameters *W*_*ij*_ and decay rates Γ_*↓*_, *κ* as in Fig. 2.

In Fig. 4d, we see periodic yield modification in Δ*G* with period ~ *ℏω*_vib_, which decays for large Δ*G*/*ℏω*_vib_ due to concomitant decline in FC factor for large changes in the number of vibrational quanta between the initial and final states. Outside of the cavity, we only see the first fringe (Fig. 4c). To observe the full periodic structure in yield, we need a large occupation of higher vibrational states which requires very large pumping rates outside of the cavity. However, under polariton condensation, the macroscopic population of the lower polariton enables these interesting periodic features to be observed at room temperature with experimentally attainable pumping rates. Additionally, polariton condensation not only modifies the reaction yield under symmetric light-matter coupling strengths, as seen in Fig. 4, it also changes the yield when the reactant and product asymmetrically couple with light (Fig. 5).

**Fig. 6:**
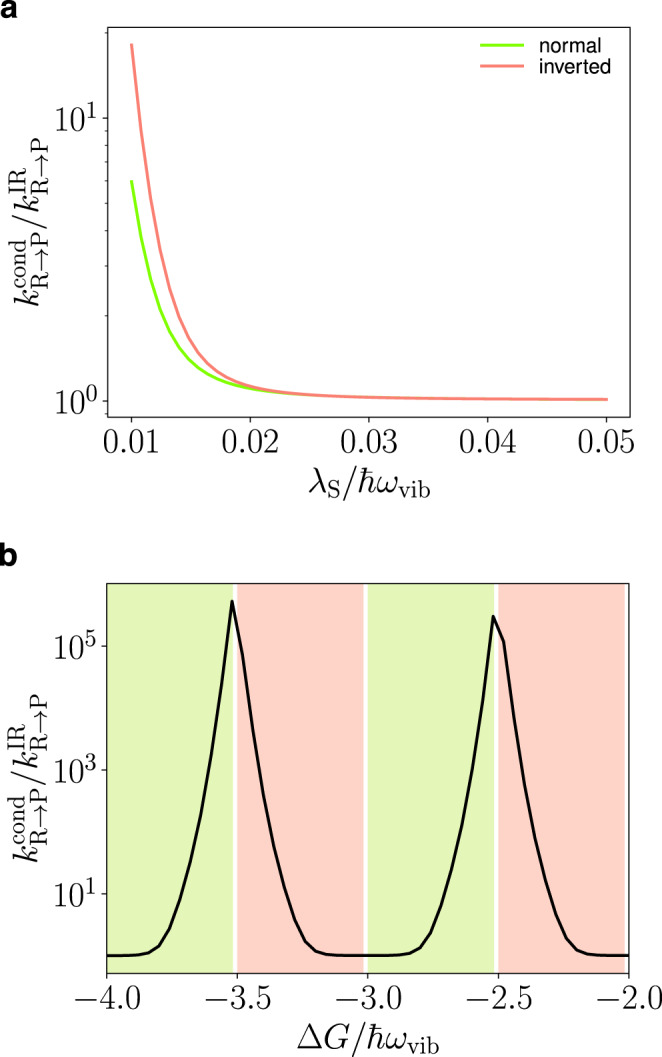
Rate constant. Ratio of the rate constants inside ${k}_{{{{{{{{\rm{R}}}}}}}}\to {{{{{{{\rm{P}}}}}}}}}^{{{{{{{{\rm{cond}}}}}}}}}$ and outside ${k}_{{{{{{{{\rm{R}}}}}}}}\to {{{{{{{\rm{P}}}}}}}}}^{{{{{{{{\rm{IR}}}}}}}}}$ of the cavity under laser excitation with Δ = −0.0695*ω*_vib_, *k*_B_*T* = 0.1389ℏ*ω*_vib_ (*T* = 298 K when ℏ*ω*_vib_ = 185 meV), *S* = 3.5, $2g\sqrt{N}=0.1{\omega }_{{{{{{{{\rm{vib}}}}}}}}}$, *P*_−_ = 0.08*N*Γ_*↓*_ and *N* = 10^7^ for symmetric coupling *g*_R_ = *g*_P_ = *g*. **a** Relative rate constant as a function of reorganization energy, *λ*_S_, with Δ*G* = −3.33ℏ*ω*_vib_ (the pink curve; here, the dominant channel lies in the inverted regime) and Δ*G* = −3.73ℏ*ω*_vib_ (the green curve; here, the dominant channel lies in the normal regime) and **b** as a function of Δ*G* with *λ*_S_ = 10^−2^ℏ*ω*_vib_. Here, the pink region corresponds to the dominant channel in the inverted regime and the green to the normal regime.

Condensation provides many additional channels for the forward and backward reactions (separated by ~*ℏ*Ω/2, see red curves in Fig. 4b, showing only the forward channels at resonance Δ = 0) due to transfer of quanta between the polariton and dark modes during the reaction. Condensation speeds up the reaction when the dominant channel is either in the inverted or in the normal regime (Fig. 6b). This is the case because there are additional channels with energy both higher (benefiting the inverted regime) and lower (benefiting the normal regime) than the dominant channel (see red curves in Fig. 4b). Apart from reduced activation energy, the additional channels provided by the condensate also have large enough FC factors to affect the rate constant. Reaction channels that involve changes in the number of quanta in the LP during the reaction have significantly larger FC factors (~10^20^ times) under condensation *N*_−_ = 0.1*N* than without any pumping *N*_−_ = 0 (Fig. S1 in the supplementary information compares them). Changes in the rate constant as a function of *λ*_S_ (Fig. 6a) and Δ*G* (Fig. 6b) are large for small *λ*_S_/*ℏω*_vib_ and when Δ*G*/*ℏω*_vib_ = *n*/2 where *n* is an odd integer since activation energy effects are large for these set of parameters.

## Discussion

Our result is a first step towards understanding the effect of Bose–Einstein condensation of polaritons on chemical reactivity. We demonstrate this effect using a simple electron transfer model (MLJ) with molecular vibrations strongly coupled to light. In particular, we show that one can counteract the massive degeneracy of dark modes and enhance polaritonic effects by having a macroscopic occupation of the lower polariton mode i.e., Bose–Einstein condensation. Our results indicate that the latter is feasible for experimentally realizable pump powers and Rabi splittings, despite the close proximity in the energy of the dark state manifold with *ℏ*Ω ~*k*_B_*T*. These results can guide the choice of suitable materials for condensation under VSC. While laser driving without SC modifies the reaction yield, this change is amplified by the condensate, due to the availability of many additional reactive channels that differ in energy by ~*ℏ*Ω/2 rather than ~*ℏω*_vib_. For a wide range of parameters, we find that this leads to a periodic dependence of reaction yield as a function of Δ*G* (with period ~*ℏω*_vib_), rendering a set of originally endergonic reactions exergonic, and vice versa. These effects are substantially weaker under laser driving and highlight both the energetic (availability of additional channels with lower activation energy) and entropic (redistribution of vibrational energy from the condensate into the polariton and dark modes upon reaction) advantages of exploiting polariton condensates for reactivity. To summarize, vibrational polariton condensation offers a novel strategy to accumulate energy into a well-defined normal mode, a holy-grail in the field of vibrational dynamics that has been historically hindered by IVR. Its successful demonstration could revive hopes of “mode selective chemistry”^50^, beyond electron transfer processes. In future work, it will be interesting to explore how the studied phenomena generalize to molecular polariton condensates in different spectral ranges.

## Supplementary information

Supplementary information

## Data Availability

Datasets generated by our code are available by email upon request to the authors.

## Code availability

Computational scripts used to generate the plots in the present article are available by email upon request to the authors.

## References

[1] Lidzey DG (1998). Strong exciton–photon coupling in an organic semiconductor microcavity. Nature.

[2] Shalabney A (2015). Coherent coupling of molecular resonators with a microcavity mode. Nat. Commun..

[3] Long JP, Simpkins B (2015). Coherent coupling between a molecular vibration and fabry–perot optical cavity to give hybridized states in the strong coupling limit. ACS Photonics.

[4] Ebbesen TW (2016). Hybrid light–matter states in a molecular and material science perspective. Acc. Chem. Res..

[5] Li X, Mandal A, Huo P (2021). Cavity frequency-dependent theory for vibrational polariton chemistry. Nat. Commun..

[6] Thomas A (2019). Tilting a ground-state reactivity landscape by vibrational strong coupling. Science.

[7] Hirai K, Takeda R, Hutchison JA, Uji-i H (2020). Modulation of prins cyclization by vibrational strong coupling. Angew. Chem..

[8] Galego J, Climent C, Garcia-Vidal FJ, Feist J (2019). Cavity casimir-polder forces and their effects in ground-state chemical reactivity. Phys. Rev. X.

[9] Li TE, Nitzan A, Subotnik JE (2020). On the origin of ground-state vacuum-field catalysis: equilibrium consideration. J. Chem. Phys..

[10] Campos-Gonzalez-Angulo JA, Yuen-Zhou J (2020). Polaritonic normal modes in transition state theory. J. Chem. Phys..

[11] Proukakis, N. P, Snoke, D. W & Littlewood, P. B. *Universal Themes of Bose-Einstein Condensation* (Cambridge Univ. Press, 2017).

[12] Daskalakis K, Maier S, Murray R, Kéna-Cohen S (2014). Nonlinear interactions in an organic polariton condensate. Nat. Mater..

[13] Plumhof JD, Stöferle T, Mai L, Scherf U, Mahrt RF (2014). Room-temperature bose–einstein condensation of cavity exciton–polaritons in a polymer. Nat. Mater..

[14] Dietrich CP (2016). An exciton-polariton laser based on biologically produced fluorescent protein. Sci. Adv..

[15] Väkeväinen AI (2020). Sub-picosecond thermalization dynamics in condensation of strongly coupled lattice plasmons. Nat. Commun..

[16] Zasedatelev AV (2019). A room-temperature organic polariton transistor. Nat. Photon..

[17] Zeb, M. A., Kirton, P. G. & Keeling, J. Incoherent charge transport in an organic polariton condensate. Preprint at https://arxiv.org/abs/2004.09790 (2020).

[18] Moore M, Vardi A (2002). Bose-enhanced chemistry: amplification of selectivity in the dissociation of molecular bose-einstein condensates. Phys. Rev. Lett..

[19] Heinzen D, Wynar R, Drummond P, Kheruntsyan K (2000). Superchemistry: dynamics of coupled atomic and molecular bose-einstein condensates. Phys. Rev. Lett..

[20] Keeling J, Kéna-Cohen S (2020). Bose–einstein condensation of exciton-polaritons in organic microcavities. Annu. Rev. Phys. Chem..

[21] Du M, Ribeiro RF, Yuen-Zhou J (2019). Remote control of chemistry in optical cavities. Chem.

[22] Delor M (2014). Toward control of electron transfer in donor-acceptor molecules by bond-specific infrared excitation. Science.

[23] Hammes-Schiffer S, Tully JC (1995). Vibrationally enhanced proton transfer. J. Phys. Chem..

[24] Strashko A, Kirton P, Keeling J (2018). Organic polariton lasing and the weak to strong coupling crossover. Phys. Rev. Lett..

[25] Bittner ER, Silva C (2012). Estimating the conditions for polariton condensation in organic thin-film microcavities. J. Chem. Phys..

[26] del Pino J, Feist J, Garcia-Vidal FJ (2015). Quantum theory of collective strong coupling of molecular vibrations with a microcavity mode. New J. Phys..

[27] Somaschi N (2011). Ultrafast polariton population build-up mediated by molecular phonons in organic microcavities. Appl. Phys. Lett..

[28] Dunkelberger A, Spann B, Fears K, Simpkins B, Owrutsky J (2016). Modified relaxation dynamics and coherent energy exchange in coupled vibration-cavity polaritons. Nat. Commun..

[29] Xiang B (2019). State-selective polariton to dark state relaxation dynamics. J. Phys. Chem. A.

[30] Fröhlich H (1968). Bose condensation of strongly excited longitudinal electric modes. Phys. Lett. A.

[31] Zhang Z, Agarwal GS, Scully MO (2019). Quantum fluctuations in the fröhlich condensate of molecular vibrations driven far from equilibrium. Phys. Rev. Lett..

[32] Banyai L, Gartner P, Schmitt O, Haug H (2000). Condensation kinetics for bosonic excitons interacting with a thermal phonon bath. Phys. Rev. B.

[33] Imamoglu A (1996). Nonequilibrium condensates and lasers without inversion: exciton-polariton lasers. Phys. Rev. A.

[34] Vurgaftman I, Simpkins BS, Dunkelberger AD, Owrutsky JC (2020). Negligible effect of vibrational polaritons on chemical reaction rates via the density of states pathway. J. Phys. Chem. Lett..

[35] Del Pino J, Garcia-Vidal FJ, Feist J (2016). Exploiting vibrational strong coupling to make an optical parametric oscillator out of a raman laser. Phys. Rev. Lett..

[36] Herrera F, Spano FC (2016). Cavity-controlled chemistry in molecular ensembles. Phys. Rev. Lett..

[37] Semenov A, Nitzan A (2019). Electron transfer in confined electromagnetic fields. J. Chem. Phys..

[38] Campos-Gonzalez-Angulo JA, Ribeiro RF, Yuen-Zhou J (2019). Resonant catalysis of thermally activated chemical reactions with vibrational polaritons. Nat. Commun..

[39] Phuc NT, Trung PQ, Ishizaki A (2020). Controlling the nonadiabatic electron-transfer reaction rate through molecular-vibration polaritons in the ultrastrong coupling regime. Sci. Rep..

[40] Marcus RA (1964). Chemical and electrochemical electron-transfer theory. Annu. Rev. Phys. Chem..

[41] Levich V (1966). Present state of the theory of oxidation-reduction in solution (bulk and electrode reactions). Adv. Electrochem. Electrochem. Eng.

[42] Jortner J (1976). Temperature dependent activation energy for electron transfer between biological molecules. J. Chem. Phys..

[43] Nesbitt DJ, Field RW (1996). Vibrational energy flow in highly excited molecules: role of intramolecular vibrational redistribution. J. Phys. Chem..

[44] Strashko A, Keeling J (2016). Raman scattering with strongly coupled vibron-polaritons. Phys. Rev. A.

[45] Miller JR, Calcaterra L, Closs G (1984). Intramolecular long-distance electron transfer in radical anions. the effects of free energy and solvent on the reaction rates. J. Am. Chem. Soc.

[46] Roche M (1990). On the polyatomic franck-condon factors. Chem. Phys. Lett..

[47] Sharp T, Rosenstock H (1964). Franck-condon factors for polyatomic molecules. J. Chem. Phys..

[48] Toniolo A, Persico M (2001). Efficient calculation of franck–condon factors and vibronic couplings in polyatomics. J. Comput. Chem..

[49] Whittaker, E. T & Watson, G. N. *A Course of Modern Analysis* 4th edn (Cambridge Univ. Press, 1996).

[50] Frei H, Pimentel GC (1985). Infrared induced photochemical processes in matrices. Annu. Rev. Phys. Chem..

