# Supplementary material for: Driving chemical reactions with polariton condensates
Source: Nat Commun. 2022 Mar 28;13:1645. doi: 10.1038/s41467-022-29290-9 (PMC8960839; doi:10.1038/s41467-022-29290-9)
Supplement: Supplementary file 1 — Supplementary information [file 41467_2022_29290_MOESM1_ESM.pdf]

Supplementary Information: Driving chemical reactions with polariton condensates

Sindhana Pannir-Sivajothi,[1] Jorge A. Campos-Gonzalez-Angulo,[1] Luis A. Martínez-Martínez,[1] Shubham Sinha,[2] and Joel Yuen-Zhou[1,*]

[1]Department of Chemistry and Biochemistry, University of California San Diego, La Jolla, California 92093, USA
[2]Department of Mathematics, University of California San Diego, La Jolla, California 92093, USA

Supplementary Note 1

We use a generalization of the dark state basis introduced in [1, 2] to reduce the number of vibrational modes involved in the reaction. The bosonic operator for $D_{R,c}$, the dark mode highly localized in the cth molecule when it is in electronic state $|R\rangle$ with total number of reactants N_R and products N_P is

$$\begin{aligned}\hat{a}_D^{(R,c)} =& \sqrt{\frac{g_P^2 N_P + g_R^2(N_R-1)}{g_P^2 N_P + g_R^2 N_R}}\hat{a}_{R,c} - \frac{g_R^2}{\sqrt{g_P^2 N_P + g_R^2 N_R}}\frac{1}{\sqrt{g_P^2 N_P + g_R^2(N_R-1)}}\sum_{i\neq c}^{N_R}\hat{a}_{R,i}\\ &- \frac{g_R g_P}{\sqrt{g_P^2 N_P + g_R^2 N_R}}\frac{1}{\sqrt{g_P^2 N_P + g_R^2(N_R-1)}}\sum_{j=1}^{N_P}\hat{a}_{P,j}.\end{aligned} \tag{S1}$$

Notice that, when $g_P = 0$, this dark mode will not involve vibrations in the product molecules and when $g_R = 0$, this dark mode will be the same as a vibration localized in the cth molecule $\hat{a}_D^{(R,c)} = \hat{a}_{R,c}$. Similarly, the bosonic operator for $D'_{P,c}$, the dark mode highly localized in the cth molecule after it has reacted and is in electronic state $|P\rangle$ with total number of reactants $N_R - 1$ and products $N_P + 1$ is

$$\begin{aligned}\hat{a}_D^{(P,c)\prime} =& \sqrt{\frac{g_P^2 N_P + g_R^2(N_R-1)}{g_P^2(N_P+1) + g_R^2(N_R-1)}}\hat{a}_{P,c} - \frac{g_R g_P}{\sqrt{g_P^2(N_P+1) + g_R^2(N_R-1)}}\frac{1}{\sqrt{g_P^2 N_P + g_R^2(N_R-1)}}\sum_{i=1}^{N_R-1}\hat{a}_{R,i}\\ &- \frac{g_P^2}{\sqrt{g_P^2(N_P+1) + g_R^2(N_R-1)}}\frac{1}{\sqrt{g_P^2 N_P + g_R^2(N_R-1)}}\sum_{j\neq c}^{N_P+1}\hat{a}_{P,j}.\end{aligned} \tag{S2}$$

Here, when $g_P = 0$, the dark mode in equation (S2) will be the same as a vibration localized in the cth molecule $\hat{a}_D^{(P,c)\prime} = \hat{a}_{P,c}$ and when $g_R = 0$, this dark mode will not involve vibrations in the reactant molecules.

Supplementary Note 2

We use Boltzmann rate equations as in [3, 4] to model polariton relaxation, and solve for the steady state of $N+1$ coupled differential equations. We assume that the scattering rate W_{ij} between polariton and dark modes is the same for all dark modes, labeled by k, this gives $W_{D_k+} = W_{D+}$ and $W_{-D_k} = W_{-D}$ [5]. Since our interests lie in the distribution of energy between polariton and dark modes rather than individual dark modes, we can simplify the problem by summing over all dark-mode equations and considering only their total population $n_D = \sum_{k=2}^{N} n_D^k$. We have the following rate equations for populations in the lower n_-, upper n_+ polaritons and all dark modes n_D,

$$\begin{aligned}\frac{dn_-}{dt} &= R_{-D} + R_{-+} - \gamma_- n_- + P_-,\\ \frac{dn_D}{dt} &= -R_{-D} + R_{D+} - \gamma_D^k n_D,\\ \frac{dn_+}{dt} &= -R_{-+} - R_{D+} - \gamma_+ n_+,\end{aligned} \tag{S3}$$

* joelyuen@ucsd.edu

where

$$\begin{aligned} R_{-\mathrm{D}} =& W_{-\mathrm{D}_k}\Big(n_{\mathrm{D}}(1+n_-) - e^{-\beta\hbar(\Omega-\Delta)/2}(N-1+n_{\mathrm{D}})n_-\Big), \\ R_{-+} =& W_{-+}\Big(n_+(1+n_-) - e^{-\beta\hbar\Omega}(1+n_+)n_-\Big), \\ R_{\mathrm{D}+} =& W_{\mathrm{D}_k+}\Big(n_+(N-1+n_{\mathrm{D}}) - e^{-\beta\hbar(\Omega+\Delta)/2}(1+n_+)n_{\mathrm{D}}\Big). \end{aligned} \tag{S4}$$

The rate coefficients can be expressed as $W_{ij} = \alpha(1+n(\beta E_{ji}))$ and $W_{ji} = \alpha n(\beta E_{ji})$ where α is a temperature independent constant, $n(\beta E_{ji})$ is the average Bose-Einstein population at energy $E_{ji} = E_j - E_i$ where $E_j > E_i$ and inverse temperature $\beta = 1/k_{\mathrm{B}}T$. The rate coefficients W_{ij} should also depend on the low-frequency vibrational density of states, for simplicity, we take the spectral density to be flat.

For all calculations in the main manuscript, we use $\kappa = \Gamma_\downarrow$, $N = 10^7$ and $\alpha = 4.33\times10^{-6}\Gamma_\downarrow$ which corresponds to $(N-1)W_{\mathrm{D}_k-} = 100\Gamma_\downarrow$ ($\sim$ 1 ps) at room temperature for light-matter coupling strength $2\hbar g\sqrt{N} = 18.5$meV at zero detuning $\Delta = 0$. These values are similar to those in experiments $\kappa = 10^{10}$ s^{-1} ($\sim$ 100 ps), $\Gamma_\downarrow = 10^{10}$ s^{-1} ($\sim$ 100 ps), and scattering from LP to all dark modes $(N-1)W_{\mathrm{D}_k-} = 10^{12}\ s^{-1}$ ($\sim$ 1 ps) [6].

Supplementary Note 3

Dependence of the FC factors on LP population is shown in Fig. S1 and they scale much better when the population in LP is large. We obtain an analytical expression for the Franck-Condon factor $|F^{f,0}_{\nu_+,\nu_-,\nu_{\mathrm{D}}}|^2$,

$$\begin{aligned} F^{f,0}_{\nu_+,\nu_-,\nu_{\mathrm{D}}} =& \langle 0, N_-, 0 | \nu_+, \nu_-, \nu_{\mathrm{D}}\rangle' \\ =& \begin{cases} \sqrt{e^{-S}S^f}\sqrt{\frac{N_-!}{\nu_+!\nu_-!\nu_{\mathrm{D}}!}}\,\tilde{w}^{-f}\left[q^{N_-}\right]\left((\tilde{x}+qx)^{\nu_+}(\tilde{y}+yq)^{\nu_-}(\tilde{z}-\tilde{z}q+zq)^{\nu_{\mathrm{D}}}\frac{\exp\left(\frac{-Swq}{1-q-wq}\right)}{(1-q-wq)^{f+1}}\right) & g_{\mathrm{P}} \neq 0 \\ (-1)^{\nu_{\mathrm{D}}}\sqrt{\frac{N_-!}{\nu_+!\nu_-!(\nu_{\mathrm{D}}-f)!}}\,(x+\tilde{x})^{\nu_+}(y+\tilde{y})^{\nu_-}u^{\nu_{\mathrm{D}}-f}\,\langle(\nu_{\mathrm{D}}-f)_{\mathrm{R},c}|(\nu_{\mathrm{D}})_{\mathrm{P},c}\rangle & g_{\mathrm{P}} = 0 \end{cases} \end{aligned} \tag{S5}$$

where $[q^n]G(q)$ is the coefficient of q^n when you expand $G(q)$ as a Taylor series, and

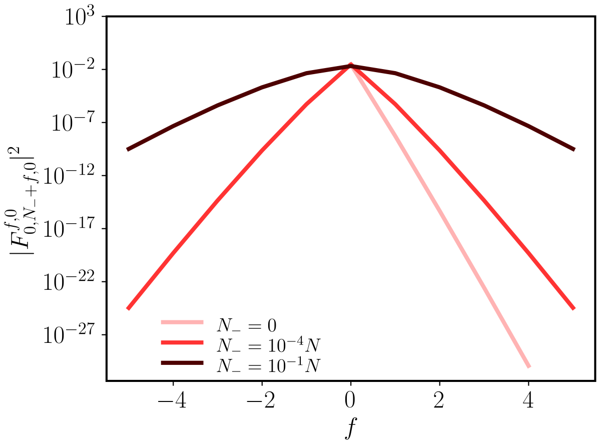

FIG. S1. **Franck-Condon (FC) factors for different channels as a function of LP population.** Reaction rate changes by the condensate occur due to channels featuring gain/loss f in vibrational quanta in the LP. The contribution of these channels is proportional to the corresponding FC factors and becomes more significant as the LP population N_- increases. Here, we show FC factors for $N_- = 0, 10^{-4}N, 10^{-1}N$ at resonance $\Delta = 0$ with symmetric light-matter coupling $2g_{\mathrm{R}}\sqrt{N} = 2g_{\mathrm{P}}\sqrt{N} = 0.1\omega_{vib}$ and Huang-Rhys factor $S = 3.5$.

$$
\begin{aligned}
u &= \left(-\cos\theta \frac{g_\mathrm{R}}{\sqrt{g_\mathrm{R}^2 N_\mathrm{R} + g_\mathrm{P}^2 N_\mathrm{P}}} \right),\\
x &= \sin\theta \cos\theta',\\
y &= \sin\theta \sin\theta',\\
z &= \left(g_\mathrm{P} \cos\theta \sqrt{\frac{g_\mathrm{R}^2 (N_\mathrm{R}-1) + g_\mathrm{P}^2 N_\mathrm{P}}{(g_\mathrm{R}^2 (N_\mathrm{R}-1) + g_\mathrm{P}^2 (N_\mathrm{P}+1))(g_\mathrm{R}^2 N_\mathrm{R} + g_\mathrm{P}^2 N_\mathrm{P})}} \right),\\
w &= \left(\frac{g_\mathrm{R} g_\mathrm{P}}{g_\mathrm{R}^2 (N_\mathrm{R}-1) + g_\mathrm{P}^2 N_\mathrm{P}} \right),\\
\tilde{x} &= \left(\frac{-\cos\theta \sin\theta' (g_\mathrm{R}^2 (N_\mathrm{R}-1) + g_\mathrm{P}^2 N_\mathrm{P})}{\sqrt{g_\mathrm{R}^2 N_\mathrm{R} + g_\mathrm{P}^2 N_\mathrm{P}} \sqrt{g_\mathrm{R}^2 (N_\mathrm{R}-1) + g_\mathrm{P}^2 (N_\mathrm{P}+1)}} \right),\\
\tilde{y} &= \left(\frac{\cos\theta \cos\theta' (g_\mathrm{R}^2 (N_\mathrm{R}-1) + g_\mathrm{P}^2 N_\mathrm{P})}{\sqrt{g_\mathrm{R}^2 N_\mathrm{R} + g_\mathrm{P}^2 N_\mathrm{P}} \sqrt{g_\mathrm{R}^2 (N_\mathrm{R}-1) + g_\mathrm{P}^2 (N_\mathrm{P}+1)}} \right),\\
\tilde{z} &= \left(-\cos\theta \frac{g_\mathrm{R}^2 (N_\mathrm{R}-1) + g_\mathrm{P}^2 N_\mathrm{P}}{g_\mathrm{P} \sqrt{g_\mathrm{R}^2 N_\mathrm{R} + g_\mathrm{P}^2 N_\mathrm{P}}} \sqrt{\frac{g_\mathrm{R}^2 (N_\mathrm{R}-1) + g_\mathrm{P}^2 N_\mathrm{P}}{g_\mathrm{R}^2 (N_\mathrm{R}-1) + g_\mathrm{P}^2 (N_\mathrm{P}+1)}} \right),\\
\tilde{w} &= \left(-\cos\theta \frac{g_\mathrm{R}^2 (N_\mathrm{R}-1) + g_\mathrm{P}^2 N_\mathrm{P}}{g_\mathrm{P} \sqrt{g_\mathrm{R}^2 N_\mathrm{R} + g_\mathrm{P}^2 N_\mathrm{P}}} \right).
\end{aligned} \tag{S6}
$$

We arrived at the expression in equation (S5) by using generating functions and the Lagrange-Bürmann formula [7]. We then recursively compute $F^{f,n}_{\nu_+,\nu_-,\nu_\mathrm{D}}$ from $F^{f,0}_{\nu_+,\nu_-,\nu_\mathrm{D}}$ [8]. Using

$$
\begin{bmatrix} \hat{a}_+ \\ \hat{a}_- \\ \hat{a}_\mathrm{D}^{(\mathrm{R},c)} \end{bmatrix} = \begin{bmatrix} J_{11} & J_{12} & J_{13} \\ J_{21} & J_{22} & J_{23} \\ J_{31} & J_{32} & J_{33} \end{bmatrix} \begin{bmatrix} \hat{a}'_+ \\ \hat{a}'_- \\ \hat{a}_\mathrm{D}^{(\mathrm{P},c)\prime} \end{bmatrix} + \begin{bmatrix} K_1 \\ K_2 \\ K_3 \end{bmatrix} \tag{S7}
$$

where

$$
\begin{aligned}
J_{11} &= \cos\theta \cos\theta' + \sin\theta \sin\theta' \left(\frac{g_\mathrm{R}^2 (N_\mathrm{R}-1) + g_\mathrm{P}^2 N_\mathrm{P} + g_\mathrm{R} g_\mathrm{P}}{\sqrt{g_\mathrm{R}^2 N_\mathrm{R} + g_\mathrm{P}^2 N_\mathrm{P}} \sqrt{g_\mathrm{R}^2 (N_\mathrm{R}-1) + g_\mathrm{P}^2 (N_\mathrm{P}+1)}} \right)\\
J_{12} &= \sin\theta' \cos\theta - \cos\theta' \sin\theta \left(\frac{g_\mathrm{R}^2 (N_\mathrm{R}-1) + g_\mathrm{P}^2 N_\mathrm{P} + g_\mathrm{R} g_\mathrm{P}}{\sqrt{g_\mathrm{R}^2 N_\mathrm{R} + g_\mathrm{P}^2 N_\mathrm{P}} \sqrt{g_\mathrm{R}^2 (N_\mathrm{R}-1) + g_\mathrm{P}^2 (N_\mathrm{P}+1)}} \right)\\
J_{13} &= \sin\theta \left(\frac{(g_\mathrm{R} - g_\mathrm{P}) \sqrt{g_\mathrm{R}^2 (N_\mathrm{R}-1) + g_\mathrm{P}^2 N_\mathrm{P}}}{\sqrt{g_\mathrm{R}^2 N_\mathrm{R} + g_\mathrm{P}^2 N_\mathrm{P}} \sqrt{g_\mathrm{R}^2 (N_\mathrm{R}-1) + g_\mathrm{P}^2 (N_\mathrm{P}+1)}} \right)\\
J_{21} &= \cos\theta' \sin\theta - \sin\theta' \cos\theta \left(\frac{g_\mathrm{R}^2 (N_\mathrm{R}-1) + g_\mathrm{P}^2 N_\mathrm{P} + g_\mathrm{R} g_\mathrm{P}}{\sqrt{g_\mathrm{R}^2 N_\mathrm{R} + g_\mathrm{P}^2 N_\mathrm{P}} \sqrt{g_\mathrm{R}^2 (N_\mathrm{R}-1) + g_\mathrm{P}^2 (N_\mathrm{P}+1)}} \right)\\
J_{22} &= \sin\theta \sin\theta' + \cos\theta \cos\theta' \left(\frac{g_\mathrm{R}^2 (N_\mathrm{R}-1) + g_\mathrm{P}^2 N_\mathrm{P} + g_\mathrm{R} g_\mathrm{P}}{\sqrt{g_\mathrm{R}^2 N_\mathrm{R} + g_\mathrm{P}^2 N_\mathrm{P}} \sqrt{g_\mathrm{R}^2 (N_\mathrm{R}-1) + g_\mathrm{P}^2 (N_\mathrm{P}+1)}} \right)\\
J_{23} &= -\cos\theta \left(\frac{(g_\mathrm{R} - g_\mathrm{P}) \sqrt{g_\mathrm{R}^2 (N_\mathrm{R}-1) + g_\mathrm{P}^2 N_\mathrm{P}}}{\sqrt{g_\mathrm{R}^2 N_\mathrm{R} + g_\mathrm{P}^2 N_\mathrm{P}} \sqrt{g_\mathrm{R}^2 (N_\mathrm{R}-1) + g_\mathrm{P}^2 (N_\mathrm{P}+1)}} \right)
\end{aligned} \tag{S8}
$$

and

$$
\begin{aligned}
J_{31} &= -\sin\theta'\left(\frac{(g_{\mathrm{R}}-g_{\mathrm{P}})\sqrt{g_{\mathrm{R}}^2(N_{\mathrm{R}}-1)+g_{\mathrm{P}}^2N_{\mathrm{P}}}}{\sqrt{g_{\mathrm{R}}^2N_{\mathrm{R}}+g_{\mathrm{P}}^2N_{\mathrm{P}}}\sqrt{g_{\mathrm{R}}^2(N_{\mathrm{R}}-1)+g_{\mathrm{P}}^2(N_{\mathrm{P}}+1)}}\right)\\
J_{32} &= \cos\theta'\left(\frac{(g_{\mathrm{R}}-g_{\mathrm{P}})\sqrt{g_{\mathrm{R}}^2(N_{\mathrm{R}}-1)+g_{\mathrm{P}}^2N_{\mathrm{P}}}}{\sqrt{g_{\mathrm{R}}^2N_{\mathrm{R}}+g_{\mathrm{P}}^2N_{\mathrm{P}}}\sqrt{g_{\mathrm{R}}^2(N_{\mathrm{R}}-1)+g_{\mathrm{P}}^2(N_{\mathrm{P}}+1)}}\right)\\
J_{33} &= \left(\frac{g_{\mathrm{R}}^2(N_{\mathrm{R}}-1)+g_{\mathrm{P}}^2N_{\mathrm{P}}+g_{\mathrm{R}}g_{\mathrm{P}}}{\sqrt{g_{\mathrm{R}}^2N_{\mathrm{R}}+g_{\mathrm{P}}^2N_{\mathrm{P}}}\sqrt{g_{\mathrm{R}}^2(N_{\mathrm{R}}-1)+g_{\mathrm{P}}^2(N_{\mathrm{P}}+1)}}\right)\\
K_1 &= -\sin\theta\frac{g_{\mathrm{R}}}{\sqrt{g_{\mathrm{R}}^2N_{\mathrm{R}}+g_{\mathrm{P}}^2N_{\mathrm{P}}}}\sqrt{S}\\
K_2 &= \cos\theta\frac{g_{\mathrm{R}}}{\sqrt{g_{\mathrm{R}}^2N_{\mathrm{R}}+g_{\mathrm{P}}^2N_{\mathrm{P}}}}\sqrt{S}\\
K_3 &= -\sqrt{\frac{g_{\mathrm{R}}^2(N_{\mathrm{R}}-1)+g_{\mathrm{P}}^2N_{\mathrm{P}}}{g_{\mathrm{R}}^2N_{\mathrm{R}}+g_{\mathrm{P}}^2N_{\mathrm{P}}}}\sqrt{S}
\end{aligned}
\tag{S9}
$$

we get the recursive formula

$$
F^{f,n+1}_{\nu_+,\nu_-,\nu_{\mathrm{D}}} = \frac{1}{\sqrt{n+1}}\Big(J_{31}\sqrt{\nu_+}F^{f,n}_{\nu_+-1,\nu_-,\nu_{\mathrm{D}}} + F^{f,n}_{\nu_+,\nu_--1,\nu_{\mathrm{D}}}J_{32}\sqrt{\nu_-} + J_{33}\sqrt{\nu_{\mathrm{D}}}F^{f,n}_{\nu_+,\nu_-,\nu_{\mathrm{D}}-1} + K_3F^{f-1,n}_{\nu_+,\nu_-,\nu_{\mathrm{D}}}\Big). \tag{S10}
$$

To derive equation (S5), we write the initial and final vibrational states in terms of creation/annihilation operators for the upper, lower polaritons and dark modes

$$
F^{f,0}_{\nu_+,\nu_-,\nu_{\mathrm{D}}} = \Big\langle 0_+0_-0_{\mathrm{D}}^{(\mathrm{R},c)}\Big|\frac{\left(\hat a_-\right)^{N_-}}{\sqrt{N_-!}}\frac{\left(\hat a'^{\dagger}_+\right)^{\nu_+}}{\sqrt{\nu_+!}}\frac{\left(\hat a'^{\dagger}_-\right)^{\nu_-}}{\sqrt{\nu_-!}}\frac{\left(\hat a_{\mathrm{D}}^{(\mathrm{P},c)\prime\dagger}\right)^{\nu_{\mathrm{D}}}}{\sqrt{\nu_{\mathrm{D}}!}}\Big|0'_+0'_-0_{\mathrm{D}}^{(\mathrm{P},c)\prime}\Big\rangle. \tag{S11}
$$

Writing the polariton modes as a linear combination of the photon $\hat a_{\mathrm{ph}}$ and bright modes $\hat a_{\mathrm{B}(N_{\mathrm{R}},N_{\mathrm{P}})}$, $\hat a_{\mathrm{B}(N_{\mathrm{R}}-1,N_{\mathrm{P}}+1)}$,

$$
\begin{aligned}
F^{f,0}_{\nu_+,\nu_-,\nu_{\mathrm{D}}} =& \frac{1}{\sqrt{N_-!\nu_+!\nu_-!\nu_{\mathrm{D}}!}}\Big\langle 0_{\mathrm{ph}}0_{\mathrm{B}(N_{\mathrm{R}},N_{\mathrm{P}})}0_{\mathrm{D}}^{(\mathrm{R},c)}\Big|\Big(\sin\theta\hat a_{\mathrm{ph}}-\cos\theta\hat a_{\mathrm{B}(N_{\mathrm{R}},N_{\mathrm{P}})}\Big)^{N_-}\Big(\cos\theta'\hat a^{\dagger}_{\mathrm{ph}}+\sin\theta'\hat a^{\dagger}_{\mathrm{B}(N_{\mathrm{R}}-1,N_{\mathrm{P}}+1)}\Big)^{\nu_+}\\
&\times\Big(\sin\theta'\hat a^{\dagger}_{\mathrm{ph}}-\cos\theta'\hat a^{\dagger}_{\mathrm{B}(N_{\mathrm{R}}-1,N_{\mathrm{P}}+1)}\Big)^{\nu_-}\Big(\hat a_{\mathrm{D}}^{(\mathrm{P},c)\prime\dagger}\Big)^{\nu_{\mathrm{D}}}\Big|0_{\mathrm{ph}}0_{\mathrm{B}(N_{\mathrm{R}}-1,N_{\mathrm{P}}+1)}0_{\mathrm{D}}^{(\mathrm{P},c)\prime}\Big\rangle.
\end{aligned}
\tag{S12}
$$

Here, $\left[\hat a_{\mathrm{ph}},\hat a_{\mathrm{B}(N_{\mathrm{R}},N_{\mathrm{P}})}\right]=0$ and $\left[\hat a_{\mathrm{ph}},\hat a_{\mathrm{B}(N_{\mathrm{R}}-1,N_{\mathrm{P}}+1)}\right]=0$, so we can use the binomial theorem and collect these operators,

$$
\begin{aligned}
F^{f,0}_{\nu_+,\nu_-,\nu_{\mathrm{D}}} =& \frac{1}{\sqrt{N_-!\nu_+!\nu_-!\nu_{\mathrm{D}}!}}\sum_{l=0}^{N_-}\sum_{m=0}^{\nu_+}\sum_{n=0}^{\nu_-}\binom{N_-}{l}\binom{\nu_+}{m}\binom{\nu_-}{n}\Big\langle 0_{\mathrm{ph}}0_{\mathrm{B}(N_{\mathrm{R}},N_{\mathrm{P}})}0_{\mathrm{D}}^{(\mathrm{R},c)}\Big|\Big(\sin\theta\hat a_{\mathrm{ph}}\Big)^{l}\\
&\times\Big(-\cos\theta\hat a_{\mathrm{B}(N_{\mathrm{R}},N_{\mathrm{P}})}\Big)^{N_--l}\Big(\cos\theta'\hat a^{\dagger}_{\mathrm{ph}}\Big)^{m}\Big(\sin\theta'\hat a^{\dagger}_{\mathrm{B}(N_{\mathrm{R}}-1,N_{\mathrm{P}}+1)}\Big)^{\nu_+-m}\Big(\sin\theta'\hat a^{\dagger}_{\mathrm{ph}}\Big)^{n}\\
&\times\Big(-\cos\theta'\hat a^{\dagger}_{\mathrm{B}(N_{\mathrm{R}}-1,N_{\mathrm{P}}+1)}\Big)^{\nu_--n}\Big(\hat a_{\mathrm{D}}^{(\mathrm{P},c)\prime\dagger}\Big)^{\nu_{\mathrm{D}}}\Big|0_{\mathrm{ph}}0_{\mathrm{B}(N_{\mathrm{R}}-1,N_{\mathrm{P}}+1)}0_{\mathrm{D}}^{(\mathrm{P},c)\prime}\Big\rangle.
\end{aligned}
\tag{S13}
$$

The only non-vanishing terms in the above summation will be those with equal number of photon creation and annihilation operators $l=m+n$, since the photon mode does not get displaced during the chemical reaction, the overlap is non-zero only when the initial and final states are exactly the same. Plugging in $l=m+n$ and $\langle 0_{\mathrm{ph}}|(\hat a_{\mathrm{ph}})^{l}(\hat a^{\dagger}_{\mathrm{ph}})^{m+n}|0_{\mathrm{ph}}\rangle=(m+n)!$

$$
\begin{aligned}
F^{f,0}_{\nu_+,\nu_-,\nu_{\mathrm{D}}} =& \frac{1}{\sqrt{N_-!\nu_+!\nu_-!\nu_{\mathrm{D}}!}}\sum_{m=0}^{\nu_+}\sum_{n=0}^{\nu_-}\binom{N_-}{m+n}\binom{\nu_+}{m}\binom{\nu_-}{n}\Big(\sin\theta\Big)^{m+n}\Big(\cos\theta'\Big)^{m}\Big(\sin\theta'\Big)^{n}\\
&\times\Big(-\cos\theta\Big)^{N_--m-n}\Big(\sin\theta'\Big)^{\nu_+-m}\Big(-\cos\theta'\Big)^{\nu_--n}(m+n)!\\
&\times\Big\langle 0_{\mathrm{B}(N_{\mathrm{R}},N_{\mathrm{P}})}0_{\mathrm{D}}^{(\mathrm{R},c)}\Big|\Big(\hat a_{\mathrm{B}(N_{\mathrm{R}},N_{\mathrm{P}})}\Big)^{N_--m-n}\Big(\hat a^{\dagger}_{\mathrm{B}(N_{\mathrm{R}}-1,N_{\mathrm{P}}+1)}\Big)^{(\nu_++\nu_-)-(m+n)}\Big(\hat a_{\mathrm{D}}^{(\mathrm{P},c)\prime\dagger}\Big)^{\nu_{\mathrm{D}}}\Big|0_{\mathrm{B}(N_{\mathrm{R}}-1,N_{\mathrm{P}}+1)}0_{\mathrm{D}}^{(\mathrm{P},c)\prime}\Big\rangle
\end{aligned}
\tag{S14}
$$

Rewriting the initial bright mode $\hat{a}_{\mathrm{B}(N_\mathrm{R},N_\mathrm{P})}$, final bright mode $\hat{a}_{\mathrm{B}(N_\mathrm{R}-1,N_\mathrm{P}+1)}$, and the highly localized final dark mode $\hat{a}_{\mathrm{D}}^{(\mathrm{P},c)\prime}$ in terms of a bright mode involving all molecules other than the reacting molecule $\hat{a}_{\mathrm{B}(N_\mathrm{R}-1,N_\mathrm{P})} = \frac{1}{\sqrt{g_\mathrm{R}^2(N_\mathrm{R}-1)+g_\mathrm{P}^2 N_\mathrm{P}}}\Big[g_\mathrm{R}\sum_{i=1}^{N_\mathrm{R}-1} a_{\mathrm{R},i} + g_\mathrm{P}\sum_{j=1}^{N_\mathrm{P}} a_{\mathrm{P},j}\Big]$, and vibrational modes of this molecule $\hat{a}_{\mathrm{P},c}$ and $\hat{a}_{\mathrm{R},c}$ we obtain

$$
\begin{aligned}
F^{f,0}_{\nu_+,\nu_-,\nu_\mathrm{D}} =& \frac{1}{\sqrt{N_-!\nu_+!\nu_-!\nu_\mathrm{D}!}} \sum_{m=0}^{\nu_+}\sum_{n=0}^{\nu_-} \binom{N_-}{m+n}\binom{\nu_+}{m}\binom{\nu_-}{n}\Big(\sin\theta\Big)^{m+n}\Big(\cos\theta'\Big)^{m}\Big(\sin\theta'\Big)^{n} \\
&\times\Big(-\cos\theta\Big)^{N_--m-n}\Big(\sin\theta'\Big)^{\nu_+-m}\Big(-\cos\theta'\Big)^{\nu_--n}(m+n)! \\
&\times\langle 0_{\mathrm{B}(N_\mathrm{R}-1,N_\mathrm{P})}0_{\mathrm{R},c}|\left[\sqrt{\frac{g_\mathrm{R}^2(N_\mathrm{R}-1)+g_\mathrm{P}^2N_\mathrm{P}}{g_\mathrm{R}^2N_\mathrm{R}+g_\mathrm{P}^2N_\mathrm{P}}}\hat{a}_{\mathrm{B}(N_\mathrm{R}-1,N_\mathrm{P})} + \frac{g_\mathrm{R}}{\sqrt{g_\mathrm{R}^2N_\mathrm{R}+g_\mathrm{P}^2N_\mathrm{P}}}\hat{a}_{\mathrm{R},c}\right]^{N_--m-n} \\
&\times\left[\sqrt{\frac{g_\mathrm{R}^2(N_\mathrm{R}-1)+g_\mathrm{P}^2N_\mathrm{P}}{g_\mathrm{R}^2(N_\mathrm{R}-1)+g_\mathrm{P}^2(N_\mathrm{P}+1)}}\hat{a}^\dagger_{\mathrm{B}(N_\mathrm{R}-1,N_\mathrm{P})} + \frac{g_\mathrm{P}}{\sqrt{g_\mathrm{R}^2(N_\mathrm{R}-1)+g_\mathrm{P}^2(N_\mathrm{P}+1)}}\hat{a}^\dagger_{\mathrm{P},c}\right]^{(\nu_++\nu_-)-(m+n)} \\
&\times\left[-\frac{g_\mathrm{P}}{\sqrt{g_\mathrm{R}^2(N_\mathrm{R}-1)+g_\mathrm{P}^2(N_\mathrm{P}+1)}}\hat{a}^\dagger_{\mathrm{B}(N_\mathrm{R}-1,N_\mathrm{P})} + \sqrt{\frac{g_\mathrm{R}^2(N_\mathrm{R}-1)+g_\mathrm{P}^2N_\mathrm{P}}{g_\mathrm{R}^2(N_\mathrm{R}-1)+g_\mathrm{P}^2(N_\mathrm{P}+1)}}\hat{a}^\dagger_{\mathrm{P},c}\right]^{\nu_\mathrm{D}} |0_{\mathrm{B}(N_\mathrm{R}-1,N_\mathrm{P})}0_{\mathrm{P},c}\rangle .
\end{aligned}
\tag{S15}
$$

Since $\left[\hat{a}_{\mathrm{B}(N_\mathrm{R}-1,N_\mathrm{P})},\hat{a}_{\mathrm{R},c}\right]=0$ and $\left[\hat{a}_{\mathrm{B}(N_\mathrm{R}-1,N_\mathrm{P})},\hat{a}_{\mathrm{P},c}\right]=0$, we can use the binomial expansion again

$$
\begin{aligned}
F^{f,0}_{\nu_+,\nu_-,\nu_\mathrm{D}} =& \frac{1}{\sqrt{N_-!\nu_+!\nu_-!\nu_\mathrm{D}!}} \sum_{m=0}^{\nu_+}\sum_{n=0}^{\nu_-}\sum_{r=0}^{N_--m-n}\sum_{p=0}^{(\nu_++\nu_-)-(m+n)}\sum_{q=0}^{\nu_\mathrm{D}} \binom{N_-}{m+n}\binom{\nu_+}{m}\binom{\nu_-}{n}\binom{N_--m-n}{r}\binom{(\nu_++\nu_-)-(m+n)}{p} \\
&\times\binom{\nu_\mathrm{D}}{q}\Big(\sin\theta\Big)^{m+n}\Big(\cos\theta'\Big)^{m}\Big(\sin\theta'\Big)^{n}(m+n)!\Big(-\cos\theta\Big)^{N_--m-n}\Big(\sin\theta'\Big)^{\nu_+-m}\Big(-\cos\theta'\Big)^{\nu_--n}(-1)^q \\
&\times\left[\frac{g_\mathrm{P}}{\sqrt{g_\mathrm{R}^2(N_\mathrm{R}-1)+g_\mathrm{P}^2(N_\mathrm{P}+1)}}\right]^{(\nu_++\nu_-+q)-(m+n+p)}\left[\sqrt{\frac{g_\mathrm{R}^2(N_\mathrm{R}-1)+g_\mathrm{P}^2N_\mathrm{P}}{g_\mathrm{R}^2(N_\mathrm{R}-1)+g_\mathrm{P}^2(N_\mathrm{P}+1)}}\right]^{\nu_\mathrm{D}+p-q} \\
&\times\left[\sqrt{\frac{g_\mathrm{R}^2(N_\mathrm{R}-1)+g_\mathrm{P}^2N_\mathrm{P}}{g_\mathrm{R}^2N_\mathrm{R}+g_\mathrm{P}^2N_\mathrm{P}}}\right]^{r}\left[\frac{g_\mathrm{R}}{\sqrt{g_\mathrm{R}^2N_\mathrm{R}+g_\mathrm{P}^2N_\mathrm{P}}}\right]^{N_--m-n-r}\langle 0_{\mathrm{B}(N_\mathrm{R}-1,N_\mathrm{P})}0_{\mathrm{R},c}|\Big(\hat{a}_{\mathrm{B}(N_\mathrm{R}-1,N_\mathrm{P})}\Big)^{r}\Big(\hat{a}_{\mathrm{R},c}\Big)^{N_--m-n-r} \\
&\times\Big(\hat{a}^\dagger_{\mathrm{B}(N_\mathrm{R}-1,N_\mathrm{P})}\Big)^{p+q}\Big(\hat{a}^\dagger_{\mathrm{P},c}\Big)^{(\nu_++\nu_-+\nu_\mathrm{D})-(m+n+p+q)}|0_{\mathrm{B}(N_\mathrm{R}-1,N_\mathrm{P})}0_{\mathrm{P},c}\rangle .
\end{aligned}
\tag{S16}
$$

The vibrational modes of all molecules other than the reacting molecule are not modified by the chemical reaction, therefore, non-zero terms in the summation satisfy $r=p+q$. Plugging in $r=p+q$ and $\langle 0_{\mathrm{B}(N_\mathrm{R}-1,N_\mathrm{P})}|(\hat{a}_{\mathrm{B}(N_\mathrm{R}-1,N_\mathrm{P})})^r(\hat{a}^\dagger_{\mathrm{B}(N_\mathrm{R}-1,N_\mathrm{P})})^{p+q}|0_{\mathrm{B}(N_\mathrm{R}-1,N_\mathrm{P})}\rangle = (p+q)!$,

$$
\begin{aligned}
F^{f,0}_{\nu_+,\nu_-,\nu_\mathrm{D}} =& \frac{1}{\sqrt{N_-!\nu_+!\nu_-!\nu_\mathrm{D}!}} \sum_{m=0}^{\nu_+}\sum_{n=0}^{\nu_-}\sum_{p=0}^{(\nu_++\nu_-)-(m+n)}\sum_{q=0}^{\nu_\mathrm{D}} \binom{N_-}{m+n}\binom{\nu_+}{m}\binom{\nu_-}{n}\binom{N_--(m+n)}{p+q}\binom{\nu_\mathrm{D}}{q}\binom{(\nu_++\nu_-)-(m+n)}{p} \\
&\times\Big(\sin\theta\Big)^{m+n}\Big(\cos\theta'\Big)^{m}\Big(\sin\theta'\Big)^{n}(m+n)!(p+q)!\Big(-\cos\theta\Big)^{N_--(m+n)}\Big(\sin\theta'\Big)^{\nu_+-m}\Big(-\cos\theta'\Big)^{\nu_--n}(-1)^q \\
&\times\left[\frac{g_\mathrm{P}}{\sqrt{g_\mathrm{R}^2(N_\mathrm{R}-1)+g_\mathrm{P}^2(N_\mathrm{P}+1)}}\right]^{(\nu_++\nu_-+q)-(m+n+p)}\left[\sqrt{\frac{g_\mathrm{R}^2(N_\mathrm{R}-1)+g_\mathrm{P}^2N_\mathrm{P}}{g_\mathrm{R}^2(N_\mathrm{R}-1)+g_\mathrm{P}^2(N_\mathrm{P}+1)}}\right]^{\nu_\mathrm{D}+p-q}\left[\sqrt{\frac{g_\mathrm{R}^2(N_\mathrm{R}-1)+g_\mathrm{P}^2N_\mathrm{P}}{g_\mathrm{R}^2N_\mathrm{R}+g_\mathrm{P}^2N_\mathrm{P}}}\right]^{p+q} \\
&\times\left[\frac{g_\mathrm{R}}{\sqrt{g_\mathrm{R}^2N_\mathrm{R}+g_\mathrm{P}^2N_\mathrm{P}}}\right]^{N_--(m+n+p+q)}\langle 0_{\mathrm{R},c}|\Big(\hat{a}_{\mathrm{R},c}\Big)^{N_--(m+n+p+q)}\Big(\hat{a}^\dagger_{\mathrm{P},c}\Big)^{(\nu_++\nu_-+\nu_\mathrm{D})-(m+n+p+q)}|0_{\mathrm{P},c}\rangle
\end{aligned}
\tag{S17}
$$

Changing variables from $\{m,n,q,p\}$ to $\{i,j,k,h\}$, where $i=\nu_+-m$, $j=\nu_--n$, $k=\nu_\mathrm{D}-q$, $h=N_--m-n-p-q$ and

$f=\nu_{+}+\nu_{-}+\nu_{\rm D}-N_{-}$,

$$F^{f,0}_{\nu_+,\nu_-,\nu_{\rm D}}=\sqrt{\frac{N_-!}{\nu_+!\nu_-!\nu_{\rm D}!}}\left(-\cos\theta\frac{g_{\rm R}^2(N_{\rm R}-1)+g_{\rm P}^2N_{\rm P}}{g_{\rm P}\sqrt{g_{\rm R}^2N_{\rm R}+g_{\rm P}^2N_{\rm P}}}\right)^{-f}\sum_{i=0}^{\nu_+}\sum_{j=0}^{\nu_-}\sum_{k=0}^{\nu_{\rm D}}\sum_{h=k-f}^{i+j+k-f}\binom{\nu_+}{i}\binom{\nu_-}{j}\binom{i+j}{h+f-k}\binom{\nu_{\rm D}}{k}$$
$$\times\left(\frac{g_{\rm R}g_{\rm P}}{g_{\rm R}^2(N_{\rm R}-1)+g_{\rm P}^2N_{\rm P}}\right)^h\left(\sin\theta\cos\theta'\right)^{\nu_+-i}\left(\frac{-\cos\theta\sin\theta'(g_{\rm R}^2(N_{\rm R}-1)+g_{\rm P}^2N_{\rm P})}{\sqrt{g_{\rm R}^2N_{\rm R}+g_{\rm P}^2N_{\rm P}}\sqrt{g_{\rm R}^2(N_{\rm R}-1)+g_{\rm P}^2(N_{\rm P}+1)}}\right)^i\left(\sin\theta\sin\theta'\right)^{\nu_--j}$$
$$\times\left(\frac{\cos\theta\cos\theta'(g_{\rm R}^2(N_{\rm R}-1)+g_{\rm P}^2N_{\rm P})}{\sqrt{g_{\rm R}^2N_{\rm R}+g_{\rm P}^2N_{\rm P}}\sqrt{g_{\rm R}^2(N_{\rm R}-1)+g_{\rm P}^2(N_{\rm P}+1)}}\right)^j\left(g_{\rm P}\cos\theta\sqrt{\frac{g_{\rm R}^2(N_{\rm R}-1)+g_{\rm P}^2N_{\rm P}}{(g_{\rm R}^2(N_{\rm R}-1)+g_{\rm P}^2(N_{\rm P}+1))(g_{\rm R}^2N_{\rm R}+g_{\rm P}^2N_{\rm P})}}\right)^{\nu_{\rm D}-k}$$
$$\times\left(-\cos\theta\frac{g_{\rm R}^2(N_{\rm R}-1)+g_{\rm P}^2N_{\rm P}}{g_{\rm P}\sqrt{g_{\rm R}^2N_{\rm R}+g_{\rm P}^2N_{\rm P}}}\sqrt{\frac{g_{\rm R}^2(N_{\rm R}-1)+g_{\rm P}^2N_{\rm P}}{g_{\rm R}^2(N_{\rm R}-1)+g_{\rm P}^2(N_{\rm P}+1)}}\right)^k\frac{1}{h!}\langle 0_{{\rm R},c}|\left(\hat a_{{\rm R},c}\right)^h\left(\hat a^\dagger_{{\rm P},c}\right)^{h+f}|0_{{\rm P},c}\rangle \tag{S18}$$

The above expression is valid only when $g_{\rm P}\neq 0$. The case of $g_{\rm P}=0$ is explained in Subsection *1*. Remembering the definitions of variables x,y,z,w and $\tilde x,\tilde y,\tilde z$ from equation (S6),

$$F^{f,0}_{\nu_+,\nu_-,\nu_{\rm D}}=\sqrt{\frac{N_-!}{\nu_+!\nu_-!\nu_{\rm D}!}}\tilde w^{-f}\sum_{i=0}^{\nu_+}\sum_{j=0}^{\nu_-}\sum_{k=0}^{\nu_{\rm D}}\sum_{h=k-f}^{i+j+k-f}\binom{\nu_+}{i}\binom{\nu_-}{j}\binom{i+j}{h+f-k}\binom{\nu_{\rm D}}{k}x^{\nu_+-i}y^{\nu_--j}z^{\nu_{\rm D}-k}\tilde x^i\tilde y^j\tilde z^k w^h$$
$$\times\sqrt{\frac{(h+f)!}{h!}}\langle h_{{\rm R},c}|(h+f)_{{\rm P},c}\rangle \tag{S19}$$

This is not easy to evaluate because of the summation over h involves the term $\langle h_{{\rm R},c}|(h+f)_{{\rm P},c}\rangle$. Substituting $\langle h_{{\rm R},c}|(h+f)_{{\rm P},c}\rangle=\sqrt{\frac{(h+f)!}{h!}}\sqrt{\frac{e^{-S}}{S^f}}\hat O_f(S)\sum_{u=0}^{h}\binom{h}{u}\frac{(-S)^u}{u!}$,

$$F^{f,0}_{\nu_+,\nu_-,\nu_{\rm D}}=\sqrt{\frac{N_-!}{\nu_+!\nu_-!\nu_{\rm D}!}}\tilde w^{-f}\sum_{i=0}^{\nu_+}\sum_{j=0}^{\nu_-}\sum_{k=0}^{\nu_{\rm D}}\sum_{h=k-f}^{i+j+k-f}\binom{\nu_+}{i}\binom{\nu_-}{j}\binom{i+j}{h+f-k}\binom{\nu_{\rm D}}{k}x^{\nu_+-i}y^{\nu_--j}z^{\nu_{\rm D}-k}\tilde x^i\tilde y^j\tilde z^k w^h$$
$$\times\frac{(h+f)!}{h!}\sqrt{\frac{e^{-S}}{S^f}}\hat O_f(S)\sum_{u=0}^{h}\binom{h}{u}\frac{(-S)^u}{u!}. \tag{S20}$$

Rewriting $(h+f)!w^h/h!$ as $\hat T_f(w)w^h$,

$$F^{f,0}_{\nu_+,\nu_-,\nu_{\rm D}}=\sqrt{\frac{e^{-S}}{S^f}}\hat O_f(S)\sqrt{\frac{N_-!}{\nu_+!\nu_-!\nu_{\rm D}!}}\tilde w^{-f}\sum_{i=0}^{\nu_+}\sum_{j=0}^{\nu_-}\sum_{k=0}^{\nu_{\rm D}}\sum_{h=k-f}^{i+j+k-f}\sum_{u=0}^{h}\binom{\nu_+}{i}\binom{\nu_-}{j}\binom{i+j}{h+f-k}\binom{\nu_{\rm D}}{k}\binom{h}{u}$$
$$\times\frac{(-S)^u}{u!}x^{\nu_+-i}y^{\nu_--j}z^{\nu_{\rm D}-k}\tilde x^i\tilde y^j\tilde z^k\hat T_f(w)w^h. \tag{S21}$$

With the operators $\hat O_f(S)$ and $\hat T_f(w)$ defined as:

$$\hat O_f(S)=\begin{cases}\left(\int dS\right)^f & f\geq 0\\ \left(\frac{d}{dS}\right)^{-f} & f<0\end{cases}$$
$$\hat T_f(w)=\begin{cases}\left(\frac{d}{dw}\right)^f w^f & f\geq 0\\ \left(\int dw\right)^{-f}w^f & f<0\end{cases} \tag{S22}$$

This simplifies to

$$
\begin{aligned}
F^{f,0}_{\nu_+,\nu_-,\nu_D} &= \sqrt{\frac{e^{-S}}{S^f}}\sqrt{\frac{N_-!}{\nu_+!\nu_-!\nu_D!}}\tilde{w}^{-f}\hat{O}_f(S)\hat{T}_f(w) \\
&\sum_{i=0}^{\nu_+}\sum_{j=0}^{\nu_-}\sum_{k=0}^{\nu_D}\sum_{h=k-f}^{i+j+k-f}\sum_{u=0}^{h}\binom{\nu_+}{i}\binom{\nu_-}{j}\binom{i+j}{h+f-k}\binom{\nu_D}{k}\binom{h}{u}\frac{(-S)^u}{u!}x^{\nu_+-i}y^{\nu_--j}z^{\nu_D-k}\tilde{x}^i\tilde{y}^j\tilde{z}^k w^h, \\
&= \sqrt{\frac{e^{-S}}{S^f}}\sqrt{\frac{N_-!}{\nu_+!\nu_-!\nu_D!}}\tilde{w}^{-f}\hat{O}_f(S)\hat{T}_f(w)M^f_{\nu_+,\nu_-,\nu_D}.
\end{aligned}
\tag{S23}
$$

Here we introduce $M^f_{\nu_+,\nu_-,\nu_D}$ as the part involving the summation in $F^{f,0}_{\nu_+,\nu_-,\nu_D}$ for more readability. Evaluating $M^f_{\nu_+,\nu_-,\nu_D}$,

$$
\begin{aligned}
M^f_{\nu_+,\nu_-,\nu_D} =& \sum_{i=0}^{\nu_+}\sum_{j=0}^{\nu_-}\sum_{k=0}^{\nu_D}\sum_{h=k-f}^{i+j+k-f}\sum_{u=0}^{h}\binom{\nu_+}{i}\binom{\nu_-}{j}\binom{i+j}{i+j+k-f-h}\binom{\nu_D}{k}\binom{h}{u}\frac{(-S)^u}{u!}x^{\nu_+-i}y^{\nu_--j}z^{\nu_D-k}\tilde{x}^i\tilde{y}^j\tilde{z}^k w^h \\
=& \sum_{i=0}^{\nu_+}\binom{\nu_+}{i}\tilde{x}^i x^{\nu_+-i}\sum_{j=0}^{\nu_-}\binom{\nu_-}{j}\tilde{y}^j y^{\nu_--j}\sum_{k=0}^{\nu_D}\binom{\nu_D}{k}\tilde{z}^k z^{\nu_D-k}\sum_{h=k-f}^{i+j+k-f}\binom{i+j}{i+j+k-f-h}w^h\sum_{u=0}^{h}\binom{h}{u}\frac{(-S)^u}{u!}
\end{aligned}
\tag{S24}
$$

We first focus on evaluating the summation over u. Without $u!$ in the denominator, this would have simply been a binomial expansion. The $u!$ makes it difficult to evaluate, but each term in the summation looks like the product of terms from a binomial $\binom{h}{u}$ and exponential $(-S)^u/u!$ expansion. We can use the powerful combinatorial technique of generating functions to evaluate the summation. Here, t is a dummy variable that we introduce to count the terms,

$$
M^f_{\nu_+,\nu_-,\nu_D} = \sum_{i=0}^{\nu_+}\binom{\nu_+}{i}\tilde{x}^i x^{\nu_+-i}\sum_{j=0}^{\nu_-}\binom{\nu_-}{j}\tilde{y}^j y^{\nu_--j}\sum_{k=0}^{\nu_D}\binom{\nu_D}{k}\tilde{z}^k z^{\nu_D-k}\sum_{h=k-f}^{i+j+k-f}\binom{i+j}{i+j+k-f-h}w^h([t^h](1+t)^h e^{-St})
\tag{S25}
$$

Each term in the summation over h is a product of coefficients of two generating functions,

$$
\begin{aligned}
M^f_{\nu_+,\nu_-,\nu_D} =& \sum_{i=0}^{\nu_+}\binom{\nu_+}{i}\tilde{x}^i x^{\nu_+-i}\sum_{j=0}^{\nu_-}\binom{\nu_-}{j}\tilde{y}^j y^{\nu_--j}\sum_{k=0}^{\nu_D}\binom{\nu_D}{k}\tilde{z}^k z^{\nu_D-k}w^{k-f}\sum_{h=k-f}^{i+j+k-f}\binom{i+j}{i+j+k-f-h}w^{h+f-k}([t^h](1+t)^h e^{-St}) \\
=& \sum_{i=0}^{\nu_+}\binom{\nu_+}{i}\tilde{x}^i x^{\nu_+-i}\sum_{j=0}^{\nu_-}\binom{\nu_-}{j}\tilde{y}^j y^{\nu_--j}\sum_{k=0}^{\nu_D}\binom{\nu_D}{k}\tilde{z}^k z^{\nu_D-k}w^{k-f}\sum_{h=k-f}^{i+j+k-f}([t^{i+j+k-f-h}](w+t)^{i+j})([t^h](1+t)^h e^{-St}).
\end{aligned}
\tag{S26}
$$

For notational convenience, let us define

$$
c_\alpha = [t^\alpha](w+t)^{i+j}, \qquad d_\beta = [t^\beta](1+t)^\beta e^{-St}.
\tag{S27}
$$

Since c_α is zero when $\alpha > i+j$, the lower limit of the summation over h can be shifted from $h=k-f$ to $h=0$,

$$
\sum_{h=k-f}^{i+j+k-f} c_{i+j+k-f-h}d_h = \sum_{h=0}^{i+j+k-f} c_{i+j+k-f-h}d_h.
\tag{S28}
$$

This summation over h can be written as the coefficient of the product of two generating function

$$
\sum_{h=0}^{i+j+k-f} c_{i+j+k-f-h}d_h = [q^{i+j+k-f}]\left(\sum_{\alpha=0}^{\infty} c_\alpha q^\alpha\right)\left(\sum_{\beta=0}^{\infty} d_\beta q^\beta\right),
\tag{S29}
$$

where q is the new dummy variable. Now we will find a closed expression for the generating function

$$
C(q) = \sum_{\alpha=0}^{\infty} c_\alpha q^\alpha, \qquad D(q) = \sum_{\beta=0}^{\infty} d_\beta q^\beta.
\tag{S30}
$$

Using binomial expansion, we get the closed expression $C(q) = (w+q)^{i+j}$. However, it is much more complicated to obtain a closed expression for $D(q)$. To do so, we use the powerful Lagrange-Bürmann formula, which states that for any generating

series $f(t)$ and $g(t)$ such that $f(0)\neq 0$ and a change of variable $q=t/f(t)$, we have an identity of generating functions in the dummy variable q,

$$\sum_{\beta=0}^{\infty}([t^{\beta}]f(t)^{\beta}g(t))q^{\beta}=\frac{g(t)}{f(t)}\frac{dt}{dq}, \tag{S31}$$

where t is expressed in terms of q. In our scenario, $f(t)=(1+t)$ and $g(t)=e^{-St}$, hence $q=t/(1+t)$, which gives us

$$t=\frac{q}{1-q} \qquad \frac{dt}{dq}=\frac{1}{(1-q)^2} \tag{S32}$$

Writing t in terms of q we get $f(t)=\frac{1}{1-q}$ and $g(t)=e^{-Sq/(1-q)}$. Therefore we have

$$\begin{aligned} D(q)&=\frac{e^{-Sq/(1-q)}}{(1-q)^2}(1-q)\\ &=\frac{e^{-Sq/(1-q)}}{(1-q)}. \end{aligned} \tag{S33}$$

Continuing with the original summation, we now have

$$M^{f}_{\nu_+,\nu_-,\nu_{\rm D}}=\sum_{i=0}^{\nu_+}\binom{\nu_+}{i}\tilde{x}^{i}x^{\nu_+-i}\sum_{j=0}^{\nu_-}\binom{\nu_-}{j}\tilde{y}^{j}y^{\nu_--j}\sum_{k=0}^{\nu_{\rm D}}\binom{\nu_{\rm D}}{k}\tilde{z}^{k}z^{\nu_{\rm D}-k}w^{k-f}\left([t^{i+j+k-f}](w+t)^{i+j}\frac{e^{-St/(1-t)}}{(1-t)}\right) \tag{S34}$$

Note that in the above expression we again replaced q with t. Repeating the calculations using Lagrange-Bürmann formula three more times, for the summation over i, j and k, we get the final compressed expression of the desired summation

$$M^{f}_{\nu_+,\nu_-,\nu_{\rm D}}=[q^{N_-}]\left((\tilde{x}+qx)^{\nu_+}(\tilde{y}+yq)^{\nu_-}\frac{(\tilde{z}-\tilde{z}q+zq)^{\nu_{\rm D}}}{(1-q)^{f}}\frac{\exp\left(\frac{-Swq}{1-q-wq}\right)}{(1-q-wq)}\right). \tag{S35}$$

Applying operators $\hat{O}_f(S)$ and $\hat{T}_f(w)$ to $M^{f}_{\nu_+,\nu_-,\nu_{\rm D}}$

$$\begin{aligned} F^{f,0}_{\nu_+,\nu_-,\nu_{\rm D}}&=\sqrt{\frac{e^{-S}}{S^f}}\sqrt{\frac{N_-!}{\nu_+!\nu_-!\nu_{\rm D}!}}\tilde{w}^{-f}[q^{N_-}]\left((\tilde{x}+qx)^{\nu_+}(\tilde{y}+yq)^{\nu_-}\frac{(\tilde{z}-\tilde{z}q+zq)^{\nu_{\rm D}}}{(1-q)^{f}}\hat{O}_f(S)\hat{T}_f(w)\frac{\exp\left(\frac{-Swq}{1-q-wq}\right)}{(1-q-wq)}\right)\\ &=\sqrt{e^{-S}S^f}\sqrt{\frac{N_-!}{\nu_+!\nu_-!\nu_{\rm D}!}}\tilde{w}^{-f}[q^{N_-}]\left((\tilde{x}+qx)^{\nu_+}(\tilde{y}+yq)^{\nu_-}(\tilde{z}-\tilde{z}q+zq)^{\nu_{\rm D}}\frac{\exp\left(\frac{-Swq}{1-q-wq}\right)}{(1-q-wq)^{f+1}}\right) \end{aligned} \tag{S36}$$

1. Product not coupled

When $g_{\rm P}=0$, the expression in equation (S36) does not apply,

$$\begin{aligned} F^{f,0}_{\nu_+,\nu_-,\nu_{\rm D}}=&\sqrt{\frac{N_-!}{\nu_+!\nu_-!\nu_{\rm D}!}}\sum_{m=0}^{\nu_+}\sum_{n=0}^{\nu_-}\sum_{q=0}^{\nu_{\rm D}}\sum_{h=\nu_{\rm D}-f-q}^{N_--m-n-q}\binom{\nu_+}{m}\binom{\nu_-}{n}\binom{(\nu_++\nu_-)-(m+n)}{h+q+f-\nu_{\rm D}}\binom{\nu_{\rm D}}{q}\\ &\times\Big(\sin\theta\Big)^{m+n}\Big(\cos\theta'\Big)^{m}\Big(\sin\theta'\Big)^{n}\Big(-\cos\theta\Big)^{N_--(m+n)}\Big(\sin\theta'\Big)^{\nu_+-m}\Big(-\cos\theta'\Big)^{\nu_--n}(-1)^{q}\\ &\times\left[\frac{g_{\rm P}}{\sqrt{g_{\rm R}^2(N_{\rm R}-1)+g_{\rm P}^2(N_{\rm P}+1)}}\right]^{(f+h+2q)-\nu_{\rm D}}\left[\sqrt{\frac{g_{\rm R}^2(N_{\rm R}-1)+g_{\rm P}^2N_{\rm P}}{g_{\rm R}^2(N_{\rm R}-1)+g_{\rm P}^2(N_{\rm P}+1)}}\right]^{\nu_{\rm D}+N_--h-m-n-2q}\\ &\times\left[\sqrt{\frac{g_{\rm R}^2(N_{\rm R}-1)+g_{\rm P}^2N_{\rm P}}{g_{\rm R}^2N_{\rm R}+g_{\rm P}^2N_{\rm P}}}\right]^{N_--h-m-n}\left[\frac{g_{\rm R}}{\sqrt{g_{\rm R}^2N_{\rm R}+g_{\rm P}^2N_{\rm P}}}\right]^{h}\sqrt{\frac{(h+f)!}{h!}}\,\langle h_{{\rm R},c}|(h+f)_{{\rm P},c}\rangle\,. \end{aligned} \tag{S37}$$

Since $g_{\rm P}=0$, only terms with $f+h+2q-\nu_{\rm D}=0$ will be non-zero,

$$\begin{aligned}F^{f,0}_{\nu_+,\nu_-,\nu_{\rm D}} =&\sqrt{\frac{N_-!}{\nu_+!\nu_-!\nu_{\rm D}!}}\sum_{m=0}^{\nu_+}\sum_{n=0}^{\nu_-}\sum_{q=0}^{\nu_{\rm D}}\binom{\nu_+}{m}\binom{\nu_-}{n}\binom{(\nu_++\nu_-)-(m+n)}{-q}\binom{\nu_{\rm D}}{q}\Big(\sin\theta\Big)^{m+n}\Big(\cos\theta'\Big)^{m}\\ &\times\Big(\sin\theta'\Big)^{n}\Big(-\cos\theta\Big)^{N_--(m+n)}\times\Big(\sin\theta'\Big)^{\nu_+-m}\Big(-\cos\theta'\Big)^{\nu_--n}(-1)^q\left(\frac{1}{\sqrt{N_{\rm R}}}\right)^{\nu_{\rm D}-f-2q}\\ &\times\left(\sqrt{\frac{N_{\rm R}-1}{N_{\rm R}}}\right)^{N_-+f+2q-\nu_{\rm D}-m-n}\sqrt{\frac{(\nu_{\rm D}-2q)!}{(\nu_{\rm D}-2q-f)!}}\,\langle(\nu_{\rm D}-2q-f)_{{\rm R},c}|(\nu_{\rm D}-2q)_{{\rm P},c}\rangle\,.\end{aligned}\tag{S38}$$

From the above, we see that $q=0$ because of the binomial coefficient involving $-q$ and

$$\begin{aligned}F^{f,0}_{\nu_+,\nu_-,\nu_{\rm D}} =&\sqrt{\frac{N_-!}{\nu_+!\nu_-!\nu_{\rm D}!}}\sum_{m=0}^{\nu_+}\sum_{n=0}^{\nu_-}\binom{\nu_+}{m}\binom{\nu_-}{n}\Big(\sin\theta\Big)^{m+n}\Big(\cos\theta'\Big)^{m}\Big(\sin\theta'\Big)^{n}\Big(-\cos\theta\Big)^{N_--(m+n)}\Big(\sin\theta'\Big)^{\nu_+-m}\\ &\times\Big(-\cos\theta'\Big)^{\nu_--n}\left(\frac{1}{\sqrt{N_{\rm R}}}\right)^{\nu_{\rm D}-f}\left(\sqrt{\frac{N_{\rm R}-1}{N_{\rm R}}}\right)^{N_-+f-\nu_{\rm D}-m-n}\sqrt{\frac{\nu_{\rm D}!}{(\nu_{\rm D}-f)!}}\,\langle(\nu_{\rm D}-f)_{{\rm R},c}|(\nu_{\rm D})_{{\rm P},c}\rangle\,.\end{aligned}\tag{S39}$$

Using the binomial expansion to collect terms, we get

$$F^{f,0}_{\nu_+,\nu_-,\nu_{\rm D}} =\sqrt{\frac{N_-!}{\nu_+!\nu_-!(\nu_{\rm D}-f)!}}(x+\tilde{x})^{\nu_+}(y+\tilde{y})^{\nu_-}u^{\nu_{\rm D}-f}\,\langle(\nu_{\rm D}-f)_{{\rm R},c}|(\nu_{\rm D})_{{\rm P},c}\rangle\,.\tag{S40}$$

2. *Reactant not coupled*

Starting from equation (S5) and substituting $g_{\rm R}=0$, we have $w=0$,

$$F^{f,0}_{\nu_+,\nu_-,\nu_{\rm D}} =\sqrt{e^{-S}S^f}\sqrt{\frac{N_-!}{\nu_+!\nu_-!\nu_{\rm D}!}}\tilde{w}^{-f}\left[q^{N_-}\right]\left((\tilde{x}+xq)^{\nu_+}(\tilde{y}+yq)^{\nu_-}(\tilde{z}-\tilde{z}q+zq)^{\nu_{\rm D}}\frac{1}{(1-q)^{f+1}}\right).\tag{S41}$$

Changing variable from q to $t=q(1+w)$

$$\begin{aligned}F^{f,0}_{\nu_+,\nu_-,\nu_{\rm D}} =&\sqrt{e^{-S}S^f}\sqrt{\frac{N_-!}{\nu_+!\nu_-!\nu_{\rm D}!}}(\tilde{w}(1+w))^{-f}[t^{N_-}]\left(\Big(\tilde{x}(1+w)+xt\Big)^{\nu_+}\Big(\tilde{y}(1+w)+yt\Big)^{\nu_-}\right.\\ &\left.\times\Big(\tilde{z}(1+w)+(z-\tilde{z})t\Big)^{\nu_{\rm D}}\frac{1}{(1-t)^{f+1}}\right)\\ =&\sqrt{e^{-S}S^f}\sqrt{\frac{N_-!}{\nu_+!\nu_-!\nu_{\rm D}!}}(\tilde{w}(1+w))^{-f}B^f_{\nu_+,\nu_-,\nu_{\rm D}},\end{aligned}\tag{S42}$$

and we call the part of $F^{f,0}_{\nu_+,\nu_-,\nu_{\rm D}}$ that involves taking the t^{N_-} coefficient

$$B^f_{\nu_+,\nu_-,\nu_{\rm D}}=[t^{N_-}]\left(\frac{G_{\nu_+,\nu_-,\nu_{\rm D}}(t)}{(1-t)^{f+1}}\right),\tag{S43}$$

where

$$G_{\nu_+,\nu_-,\nu_{\rm D}}(t)=\Big(\tilde{x}(1+w)+xt\Big)^{\nu_+}\Big(\tilde{y}(1+w)+yt\Big)^{\nu_-}\Big(\tilde{z}(1+w)+(z-\tilde{z})t\Big)^{\nu_{\rm D}}.\tag{S44}$$

When $f<0$, then $\deg(G_{\nu_+,\nu_-,\nu_{\rm D}}(t)(1-t)^{-f-1})=N_--1$, therefore, $B^f_{\nu_+,\nu_-,\nu_{\rm D}}=0$. When $f=0$, then $\deg(G_{\nu_+,\nu_-,\nu_{\rm D}}(t))=N_-$ and we have the following identity

$$[t^{N_-}]\left(\frac{G_{\nu_+,\nu_-,\nu_{\rm D}}(t)}{(1-t)}\right)=G_{\nu_+,\nu_-,\nu_{\rm D}}(1).\tag{S45}$$

Using this identity, we obtain the expression for $B^{f}_{\nu_+,\nu_-,\nu_D}$ base case $f=0$,

$$B^{0}_{\nu_+,\nu_-,\nu_D} = G_{\nu_+,\nu_-,\nu_D}(1). \tag{S46}$$

Now that we have the result for $f=0$, let's derive a recursive formula for $B^{f}_{\nu_+,\nu_-,\nu_D}$ when $f \geq 1$. The coefficient of t^{N_-+1} in a series $\sum_n a_n t^n$ is related to the coefficient of t^{N_-} of the derivative of the same series. Using this,

$$\begin{aligned} \left[t^{N_-}\right] \frac{d}{dt}\left(\frac{G_{\nu_+,\nu_-,\nu_D}(t)}{(1-t)^f}\right) &= (N_-+1)[t^{N_-+1}]\left(\frac{G_{\nu_+,\nu_-,\nu_D}(t)}{(1-t)^f}\right) \\ [t^{N_-}]\left(\frac{G'_{\nu_+,\nu_-,\nu_D}(t)}{(1-t)^f}\right) + f[t^{N_-}]\left(\frac{G_{\nu_+,\nu_-,\nu_D}(t)}{(1-t)^{f+1}}\right) &= (N_-+1)[t^{N_-+1}]\left(\frac{G_{\nu_+,\nu_-,\nu_D}(t)}{(1-t)^f}\right) \\ fB^{f}_{\nu_+,\nu_-,\nu_D} &= (N_-+1)B^{f-1}_{\nu_+,\nu_-,\nu_D} - [t^{N_-}]\left(\frac{G'_{\nu_+,\nu_-,\nu_D}(t)}{(1-t)^f}\right) \end{aligned} \tag{S47}$$

$$B^{f}_{\nu_+,\nu_-,\nu_D} = \frac{1}{f}\left((N_-+1)B^{f-1}_{\nu_+,\nu_-,\nu_D} - \nu_+ x B^{f-1}_{\nu_+-1,\nu_-,\nu_D} - \nu_- y B^{f-1}_{\nu_+,\nu_--1,\nu_D} - \nu_D(z-\tilde{z})B^{f-1}_{\nu_+,\nu_-,\nu_D-1}\right) \tag{S48}$$

The expression for $B^{f}_{\nu_+,\nu_-,\nu_D}$ for all the different cases,

$$B^{f}_{\nu_+,\nu_-,\nu_D} = \begin{cases} 0 & f<0 \\ G_{\nu_+,\nu_-,\nu_D}(1) & f=0 \\ \frac{1}{f}\left((N_-+1)B^{f-1}_{\nu_+,\nu_-,\nu_D} - \nu_+ x B^{f-1}_{\nu_+-1,\nu_-,\nu_D} - \nu_- y B^{f-1}_{\nu_+,\nu_--1,\nu_D} - \nu_D(z-\tilde{z})B^{f-1}_{\nu_+,\nu_-,\nu_D-1}\right) & f>0 \end{cases} \tag{S49}$$

3. *Product and reactant equally coupled*

For the special case when $g_R = g_P$, we change variable $t = q(1+w)$ and the expression becomes

$$\begin{aligned} F^{f,0}_{\nu_+,\nu_-,\nu_D} =& \sqrt{e^{-S}S^f}\sqrt{\frac{N_-!}{\nu_+!\nu_-!\nu_D!}}(\tilde{w}(1+w))^{-f}[t^{N_-}]\left(\left(\tilde{x}(1+w)+xt\right)^{\nu_+}\left(\tilde{y}(1+w)+yt\right)^{\nu_-}\right. \\ &\left.\times\left(\tilde{z}(1+w)+(z-\tilde{z})t\right)^{\nu_D}\frac{\exp\left(\frac{-Swt}{(1+w)(1-t)}\right)}{(1-t)^{f+1}}\right) \\ =& \sqrt{e^{-S}S^f}\sqrt{\frac{Q!}{\nu_+!\nu_-!\nu_D!}}(\tilde{w}(1+w))^{-f}A^{f}_{\nu_+,\nu_-,\nu_D}, \end{aligned} \tag{S50}$$

where

$$A^{f}_{\nu_+,\nu_-,\nu_D} = [t^{N_-}]\left(\left(\tilde{x}(1+w)+xt\right)^{\nu_+}\left(\tilde{y}(1+w)+yt\right)^{\nu_-}\left(\tilde{z}(1+w)+(z-\tilde{z})t\right)^{\nu_D}\frac{\exp\left(\frac{-Swt}{(1+w)(1-t)}\right)}{(1-t)^{f+1}}\right). \tag{S51}$$

Expanding the exponential,

$$
\begin{aligned}
A^{f}_{v_+,v_-,v_\mathrm{D}} &= [t^{Q}]\left(\left(\tilde{x}(1+w)+xt\right)^{v_+}\left(\tilde{y}(1+w)+yt\right)^{v_-}\left(\tilde{z}(1+w)+(z-\tilde{z})t\right)^{v_\mathrm{D}}\frac{1}{(1-t)^{f+1}}exp\left(\frac{-Swt}{(1+w)(1-t)}\right)\right) \\
&= \sum_{n=0}^{\infty}\frac{1}{n!}[t^{Q}]\left(\left(\tilde{x}(1+w)+xt\right)^{v_+}\left(\tilde{y}(1+w)+yt\right)^{v_-}\left(\tilde{z}(1+w)+(z-\tilde{z})t\right)^{v_\mathrm{D}}\frac{1}{(1-t)^{f+1}}\left(\frac{-Swt}{(1+w)(1-t)}\right)^{n}\right) \\
&= \sum_{n=0}^{\infty}w^{n}\frac{1}{n!}\left(\frac{-S}{1+w}\right)^{n}[t^{Q}]\left(\left(\tilde{x}(1+w)+xt\right)^{v_+}\left(\tilde{y}(1+w)+yt\right)^{v_-}\left(\tilde{z}(1+w)+(z-\tilde{z})t\right)^{v_\mathrm{D}}\frac{t^{n}}{(1-t)^{f+1+n}}\right) \qquad \text{(S52)} \\
&= \sum_{n=0}^{\infty}w^{n}\frac{1}{n!}\left(\frac{-S}{1+w}\right)^{n}[t^{Q-n}]\left(\left(\tilde{x}(1+w)+xt\right)^{v_+}\left(\tilde{y}(1+w)+yt\right)^{v_-}\left(\tilde{z}(1+w)+(z-\tilde{z})t\right)^{v_\mathrm{D}}\frac{1}{(1-t)^{f+1+n}}\right) \\
&= \sum_{n=0}^{\infty}w^{n}\frac{1}{n!}\left(\frac{-S}{1+w}\right)^{n}B^{f+n}_{v_+,v_-,v_\mathrm{D}}
\end{aligned}
$$

For the case when $g_\mathrm{R}=g_\mathrm{P}$, $w=1/(N-1)$ is very small when a large number of molecules are coupled to the cavity. Therefore, the summation in $A^{f}_{v_+,v_-,v_\mathrm{D}}$ converges quickly and is easy to calculate on a computer.

Supplementary References

[1] Strashko, A. & Keeling, J. Raman scattering with strongly coupled vibron-polaritons. *Phys. Rev. A* **94**, 023843 (2016).

[2] Campos-Gonzalez-Angulo, J. A., Ribeiro, R. F. & Yuen-Zhou, J. Resonant catalysis of thermally activated chemical reactions with vibrational polaritons. *Nat. Commun.* **10**, 1–8 (2019).

[3] Banyai, L., Gartner, P., Schmitt, O. & Haug, H. Condensation kinetics for bosonic excitons interacting with a thermal phonon bath. *Phys. Rev. B* **61**, 8823 (2000).

[4] Cao, H. T., Doan, T., Thoai, D. T. & Haug, H. Condensation kinetics of cavity polaritons interacting with a thermal phonon bath. *Phys. Rev. B* **69**, 245325 (2004).

[5] del Pino, J., Feist, J. & Garcia-Vidal, F. J. Quantum theory of collective strong coupling of molecular vibrations with a microcavity mode. *New J. Phys.* **17**, 053040 (2015).

[6] Xiang, B. *et al.* State-selective polariton to dark state relaxation dynamics. *J. Phys. Chem. A* **123**, 5918–5927 (2019).

[7] Whittaker, E. T. & Watson, G. N. *A Course of Modern Analysis, 4th ed* (Cambridge Univ. Press, Cambridge, 1996).

[8] Roche, M. On the polyatomic franck-condon factors. *Chem. Phys. Lett.* **168**, 556–558 (1990).
